# Novel insights into extracellular vesicles: An update on biomolecules, immunomodulation and clinical strategies in skin melanoma

**DOI:** 10.1002/ctm2.70658

**Published:** 2026-04-16

**Authors:** Junshu Li, Wencheng Zhou, Ying Cen, Yong Qing

**Affiliations:** ^1^ Department of Burn and Plastic Surgery West China Hospital Sichuan University Chengdu China; ^2^ Department of Biotherapy Cancer Center and State Key Laboratory of Biotherapy West China Hospital Sichuan University Chengdu China; ^3^ Department of Medical Aesthetics West China School of Public Health and West China Fourth Hospital Sichuan University Chengdu China

**Keywords:** extracellular vesicles, melanoma, tumour microenvironment, biomarkers, therapeutic strategies

## Abstract

Extracellular vesicles (EVs) have become key mediators of intercellular communication in melanoma cells and significantly affect disease progression by delivering bioactive molecules. EVs are loaded with a wide variety of biomolecule carriers, including proteins, DNA, mRNAs and non‐coding RNAs, which can affect processes such as tumour growth, metastasis, angiogenesis, drug resistance and immune escape. Melanoma‐derived EVs dynamically reshape the tumour microenvironment (TME) by regulating different cellular components, such as cancer‐associated fibroblasts (CAFs), endothelial cells, T cells, macrophages and natural killer (NK) cells. In addition, we emphasize the enormous potential of EVs as minimally invasive biomarkers for diagnosis, prognosis prediction and treatment monitoring. The biocompatibility and targeting characteristics of EVs also make them promising platforms for drug delivery, including as carriers for chemotherapy drugs and immunomodulators. In this review, we emphasized the key role of EVs in melanoma and their dynamic regulation of microenvironment components, providing new diagnostic and therapeutic approaches for the use of EVs in the treatment of invasive malignant tumours.

**Highlights:**

- Melanoma‐derived EVs carry diverse biomolecules that reprogram the tumor microenvironment.
- EVs mediate immune escape, drug resistance and metastasis via key signaling pathways.
- EVs serve as minimally invasive liquid biopsy biomarkers for diagnosis, prognosis, and therapy monitoring.
- Engineered EVs offer promising platforms for targeted drug delivery and immunotherapy in melanoma.

## Graphical abstract

Extracellular vesicles mediate melanoma progression by transferring biomolecules (proteins, nucleic acids) that reshape the tumour microenvironment, promote immune evasion and drive metastasis. They also serve as diagnostic biomarkers and engineered therapeutic carriers for targeted therapy.

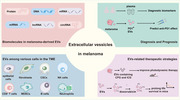

## 1 INTRODUCTION

Melanoma is a highly malignant type of tumour derived from melanocytes that poses a great threat to human health. Melanoma not only occurs on the surface of the skin, but also may arise from mucous membranes and internal organs, and has high invasiveness and migration ability.^1^ In recent years, with the deterioration of the environment and lifestyle changes, the incidence rate and mortality rate of melanoma have shown increasing trends annually.^2^ Although early melanoma can usually be cured by surgery, the disease can rapidly spread to other organs through the blood and lymphatic pathways. For advanced metastatic melanoma, treatment strategy options are very limited, and traditional radiotherapy and chemotherapy have poor efficacy.^3^ Melanoma has complex biological characteristics, especially high immune evasion ability and resistance to chemotherapy. Melanoma pathogenesis is influenced by both the tumour cells and the dynamic interactions within the surrounding tumour microenvironment (TME).^4^ TME is a complex and diverse setting comprising various cellular components such as cancer‐associated fibroblasts, vascular and lymphatic endothelial cells, as well as innate and adaptive immune cells.^5^ These cellular components reshape the extracellular matrix and affect the progression of melanoma by regulating cytokines and chemokines. Melanoma is often regarded as a highly immunogenic solid tumour, abundant in neoantigens and vulnerable to immune responses.^6^ However, the varied patient responses to immune checkpoint inhibitors like anti‐PD‐1 and anti‐CTLA‐4 reflect the intricate mechanisms of the immune microenvironment.^7^

EVs are nanoscale messengers for intercellular crosstalk and systemic landscape reconstruction. EVs are phospholipid bilayer‐enclosed particles predominantly released into the extracellular space.^8^ EVs are categorized into subtypes like exosomes, originating from the endosome system via polycystic bodies and micro‐vesicles, which are released from the plasma membrane. These classifications are based on size, synthesis pathways and content composition.^9^ EV is now considered a key molecule for intercellular communication and is capable of transporting various bioactive substances to nearby or distant receptor cells for remote regulation.^10^ EVs carry bioactive substances like proteins (receptors, enzymes, growth factors), lipids, metabolites and diverse nucleic acids (DNA fragments, mRNAs, non‐coding RNAs).^11^ Tumour cell‐derived EVs can modify the TME and influence melanoma progression, metastasis, drug resistance and immune evasion by transporting diverse nucleic acids and proteins.^12^ ^13^ For example, melanoma‐derived EVs can facilitate the transformation of normal fibroblasts into CAFs, enhance angiogenesis, deplete T cells, elevate M2 immunosuppressive macrophages and increase MDSCs, thereby suppressing tumour immunity.^14^ ^15^ In addition, the molecular characteristics of these EVs reflect the pathological state of tumour patients, making them promising candidates for liquid biopsy. The presence of extracellular vesicles in plasma provides a minimally invasive strategy for disease detection, risk prediction, treatment response monitoring and recurrence detection.^16^ ^17^

EVs have broad prospects as biomarkers in the clinical diagnosis of melanoma and as targets and drugs in tumour therapy.^18^ In terms of diagnosis, researchers have great potential in developing highly sensitive and specific detection strategies by detecting specific EV‐related proteins (such as PD‐L1), nucleic acids (such as mutated *BRAF* DNA and specific miRNA profiles) or other biomolecules.^19^, ^20^, ^21^ In terms of therapy, the biocompatibility and targeting efficiency of EVs make them complex drug delivery systems. EVs carrying chemical agents, nucleic acids (e.g., siRNAs or mRNAs) or immunostimulatory molecules from immune cells can efficiently bypass biological barriers and enhance anti‐tumour immune responses.^22^, ^23^ Using electroporation technology to encapsulate BRAF‐siRNA into mature dendritic cell‐derived exosomes and construct a nanosystem for immunotherapy of melanoma. A previous study revealed that the nanosystem can act on the immune microenvironment of melanoma, promote T lymphocyte activation and inhibit tumour progression.^24^

This review thoroughly examines the crucial role of EVs in the onset and development of cutaneous melanoma. We conducted an in‐depth analysis of the biological characteristics, biogenesis, loaded molecules and molecular mechanisms of EVs acting on receptor cells. Moreover, we highlight the multifaceted roles of EVs in modulating the TME, influencing tumour metastasis and contributing to drug resistance. Finally, we explored the potential of EVs as clinical diagnostic and prognostic biomarkers, and elucidated their innovative applications in engineering therapeutic carriers and vaccines to alleviate melanoma. This review aims to elucidate the profound impact of EV‐mediated communication on the progression of melanoma and provide new ideas for future exploration of molecular mechanisms and clinical applications.

## 2 MELANOMA

Malignant melanoma, a tumour arising from melanocytes, is marked by swift metastasis and significant drug resistance, posing a serious threat to human health.^25^ The main cause of melanoma is damage caused by ultraviolet radiation after exposure to sunlight.^26^ Ultraviolet radiation can directly cause DNA damage and create a microenvironment that promotes tumour progression.^27^ Additional melanoma risk factors encompass family history, underdeveloped pigmented nevi, gene mutations like *CDKN2A* and immune suppression.^28^ At present, the pathogenesis of melanoma still needs further research.

Melanoma development and advancement are influenced by a complex accumulation of genetic and epigenetic alterations.^29^ An important study revealed activating mutations in the *BRAF* oncogene, which occur in approximately 40–50% of skin melanomas.^30^ Most of these proteins experience constitutive activation of the MAPK signalling pathway (RAS‐RAF‐MEK‐ERK) due to the V600E substitution, a crucial driver of cell proliferation, survival and metabolism.^31^ Other common mutations that drive melanoma progression include *NRAS* (15‐20%) and *NF1* (10–15%).^32^ In addition, in many familial and sporadic cases, the tumour suppressor *CDKN2A* is inactivated and the *TERT* promoter is mutated, thereby promoting cell self‐renewal. Tumour heterogeneity occurs both between patients and within individual tumours, resulting in phenotypic diversity that can affect treatment outcomes.^33^, ^34^ Melanoma is highly malignant, often metastasizing distantly via blood and lymphatic systems, and is associated with a poor prognosis. Over 20% of individuals with melanoma develop metastasis, and the survival rate for those with metastatic melanoma is between 5% and 10%.^35^ The shift of tumour cells from the radial to the vertical growth phase suggests potential metastasis. And dysregulation of critical signalling pathways, such as PI3K/AKT, WNT/β‐catenin and MITF, facilitates tumour metastasis by collectively coordinating cell cycle progression, evasion of apoptosis and invasion.^36^, ^37^, ^38^

The most widely used treatment strategy for melanoma currently is surgical resection, whereas immune checkpoint inhibitors or targeted therapy are adjuvant treatment strategies for high‐risk patients, thereby reducing the probability of recurrence.^39^ Immune checkpoint inhibitors, including monoclonal antibodies CTLA‐4 (such as ipilimumab) and PD‐1 (such as nivolumab and pembrolizumab), have shown good efficacy and maintain long‐term survival in a few melanoma patients.^40^ At the same time, targeted therapy for melanoma involving BRAF inhibitors (such as vemurafenib and dabrafenib) and MEK inhibitors (such as trametinib and cobimetinib) provides a promising treatment strategy for patients with *BRAF* V600 mutations.^41^ Although advancements have been made in melanoma treatment, the activation of various signalling pathways and adaptation to the TME have commonly resulted in drug resistance.^42^ In summary, the strong mutation characteristics of melanoma and the dynamic changes in the TME enable tumour cells to adapt to and evade these treatments.

Notably, melanoma is considered one of the most immunogenic solid tumours and is characterized by a high burden of tumour‐specific neoantigens caused by mutations induced by ultraviolet radiation. In theory, the increase in tumour neoantigens enables tumour cells to be effectively recognized by the host immune system.^43^, ^44^ However, melanoma microenvironment establishes an immunosuppressive state through various molecular mechanisms, including upregulation of immune checkpoint molecules such as PD‐L1 on tumour and stromal cells, recruitment and activation of Tregs and MDSCs, secretion of immunosuppressive factors (such as TGF‐β, IL‐10 and VEGF), and induction of T cell depletion.^45^, ^46^, ^47^ A high proportion of melanoma patients are tolerant to immunotherapy or experience recurrence. In addition, EV secretion and phagocytosis significantly contribute to TME remodelling.^48^ Extracellular vesicles derived from melanoma cells can regulate immune escape and chemoresistance by carrying “cargo” such as RNA and proteins.^49^ Also, extracellular vesicles can usually be obtained from the blood, saliva and urine of cancer patients.^50^ Moreover, extracellular vesicles from different organs and cell sources play different roles, such as tumour‐infiltrating lymphocytes that can exert anti‐tumour effects.^51^ The complex communication network between melanoma and the microenvironment regulates tumour progression and immune tolerance, and extracellular vesicles have become key regulatory factors.

## 3 EXTRACELLULAR VESICLES (EVS): CLASSIFICATION, BIOGENESIS AND FUNCTION

### 3.1 Classification and biogenesis

**FIGURE 1:**
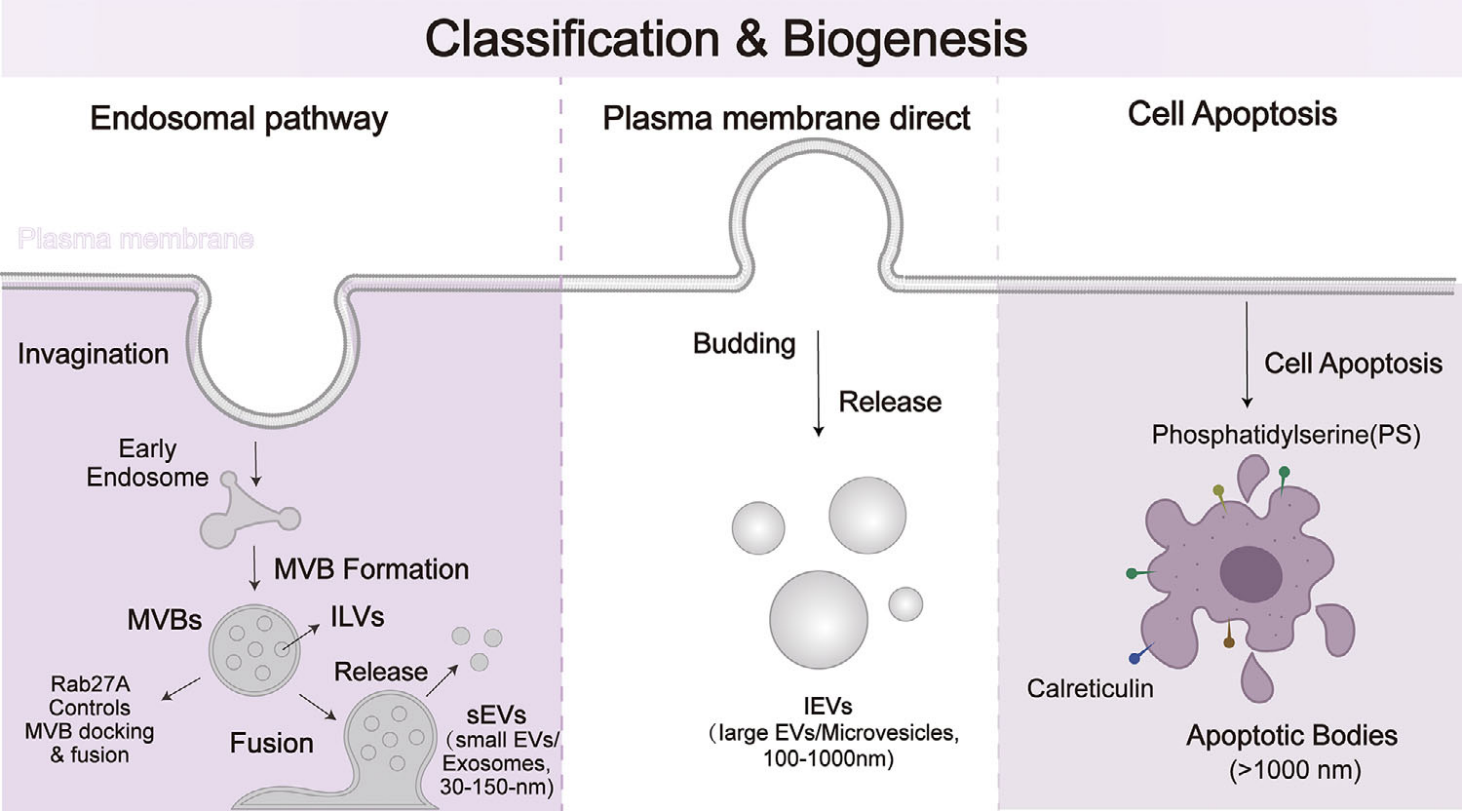
Classification and biogenesis of EVs. EVs are mainly divided into three subtypes, based on their different biological sources and sizes. Small EVs (sEVs, 30–150 nm, including exosomes) form via the endosomal pathway, involving plasma membrane invagination, early endosome maturation and the formation of intra‐luminal vesicles (ILVs) within multi‐vesicular bodies (MVBs). This process is driven by ceramide‐dependent mechanisms and concludes with MVB fusion with the plasma membrane controlled by Rab27A. Large EVs (lEVs, 100–1000 nm, including microvesicles) are generated by direct outward budding and shedding from the plasma membrane. Apoptotic bodies (>1000 nm) are released during cell death, marked by the exposure of phosphatidylserine and calreticulin on their surface.

Extracellular vesicles are bilayer lipid nanoparticles secreted by various cells into the extracellular environment and participate in intercellular information exchange.^52^ Extracellular vesicles are loaded with cellular metabolites such as nucleic acids, proteins and lipids.^53^ EVs are categorized into small EVs, large EVs and apoptotic bodies based on their biogenesis, size and molecular composition^54^ (Figure 1). Among these, small EVs (30–150 nm) originate in the endosomal system through invagination from multi‐vesicular bodies (MVBs) and are released when MVBs fuse with the plasma membrane.^55^ Large EVs (100–1000 nm) are produced through direct budding and shedding from the plasma membrane.^56^ Apoptotic bodies, which are released during programmed cell death, exceed 1000 nm in size.^57^ In recent years, research has identified spherical extracellular vesicles in both non‐metastatic and metastatic liver tissues. Under transmission electron microscopy tomography and 3D reconstruction, small spherical structures with a diameter of approximately 30–200 nm were observed.^58^ Extracellular vesicles are crucial for the malignant progression of tumours, but the molecular mechanisms underlying their secretion are still unclear.

Emerging evidence suggests that EVs from melanoma cells are highly heterogeneous, with distinct subtypes differing significantly in their biogenesis, molecular cargo and target cells.^59^ Small EVs (such as exosomes) are stable in the circulatory system due to their small size, enabling them to significantly influence the microenvironment before distal metastasis. Melanoma‐derived small EVs can present immune checkpoint molecules like PD‐L1 on their surface, directly suppressing T cell activity. Concurrently, they deliver specific signals to immune cells, such as inducing the production of immunosuppressive macrophages by upregulating CD36 on the surface of endothelial/monocytes.^60^ More importantly, the molecular cargo of sEVs can accurately reflect the cellular state. Compared with small EVs derived from normal melanocytes, mature melanocyte markers such as MART‐1 are reduced, while epithelial–mesenchymal transition (EMT)‐related adhesion molecules are significantly increased. Large EVs (such as micro‐vesicles and melanosomes) can carry more complex and larger molecular cargo, including protein complexes, metabolic enzymes and organelle components, which play a critical role in reshaping the local microenvironment in the proximal region.^61^ The melanosomes secreted by melanoma cells can be engulfed by neighbouring keratinocytes or fibroblasts, loaded with new proteins for secondary release, and then engulfed by macrophages to promote angiogenesis and tumour metastasis.^62^ In addition, apoptotic bodies expose calreticulin and carry tumour antigens, thereby monitoring somatic immunity or triggering anti‐tumour immune responses.^63^ Research on the functions of these subgroups remains limited. Future research should investigate the molecular mechanisms of EV subgroups related to metastasis and drug resistance.

Intracellular and extracellular signals regulate the formation and secretion of EVs. For instance, inhibiting cholesterol transport can lead to the internalization of Cav‐1 and the production of double‐ended vesicles, which then fuse with the cytoplasmic membrane to affect the release of extracellular vesicles, thereby maintaining cellular function and body balance.^64^ Additionally, lactic acid can reduce intracellular cAMP production, activate PKA expression and inhibit the ubiquitination of HIF‐1α, thereby promoting *Rab27A* transcription and increasing the release of tumour‐associated extracellular vesicles.^65^ A few studies have partially elucidated the molecular mechanisms controlling the fusion of MVB with the plasma membrane. The physiological mechanism of SNARE protein‐mediated MVB fusion with the plasma membrane remains highly debated. Recent studies have shown that VAMP‐7, syntaxin 4 and SNAP‐23 do not affect the synthesis and transport of MVBs, but they do affect the fusion process between MVBs and PM.^66^

EVs display considerable diversity in size, composition and function, often mirroring the physiological or pathological condition of their originating cells.^67^ Metastatic melanoma secretes large CD133^+^ extracellular vesicles that are crucial for intercellular communication and tumour progression regulation. The extracellular vesicles have a diameter of approximately 2 to 6 µm and are rich in mitochondria and lipid droplets, which are involved in the biological processes of tumour cell metabolism regulation. Meanwhile, knocking down the expression of CD133 can regulate the nuclear localization of lipid droplets and severely affect the production of large extracellular vesicles.^68^ In addition, extracellular vesicles can transfer functional receptors between various cells. In vitro experiments demonstrated that extracellular vesicles with elevated ROR1/2 expression enhanced the proliferation and migration of breast cancer cells with low ROR expression. ROR1/2, as a Wnt pathway receptor, can be delivered to the surface of ROR1/2‐negative tumour cells through extracellular vesicles and induce the acquisition of invasive phenotypes.^69^

### 3.2 Cargo sorting mechanisms

Extracellular vesicles selectively sort cargo through the synergistic action of multiple molecular mechanisms, ensuring precise encapsulation and release of information. Among them, the endosomal sorting complex required for transport (ESCRT) pathway represents the most classical sorting mechanism, which recruits cargo to the endosomal membrane by recognizing ubiquitinated tags and drives membrane invagination.^70^ In glioblastoma, EGFRvIII signalling activates the ESCRT pathway, leading to the ubiquitination and sorting of the transmembrane receptor EGFRvIII into exosomes, thereby promoting tumour growth and invasion.^71^, ^72^ Beyond proteins, the sorting of nucleic acid cargo typically relies on RNA‐binding proteins. During high oxygen stress in lung epithelial cells, phosphorylated caveolin‐1 can modify hnRNPA2B1 and enhance glycosylation of O‐GlcNAc, thereby selectively packaging miRNAs (such as miR‐17, miR‐93) into EVs.^73^ Similarly, the FMRP protein recognizes and binds miRNAs containing the AAUGC sequence during inflammation, subsequently transferring them to multi‐vesicular bodies for release via exosomes.^74^ Furthermore, cargo sorting is regulated by cellular states and microenvironments. For example, *KRAS* gene mutations induce silencing of the key protein Ago2 in the downstream MEK/Erk signalling pathway through phosphorylation and inhibit its entry into exosomes, while promoting the sorting of carcinogenic miRNAs by other RBPs.^75^, ^76^ Under hypoxic conditions, HIF‐1α can upregulate pro‐angiogenic miRNAs (such as miR‐210) and synergistically promote the sorting of RNAs and proteins into EVs by enhancing the expression of ceramide synthase *nSMase2*, thereby enhancing the adaptability and metastatic potential of tumours.^77^, ^78^

### 3.3 Functional roles in melanoma

Extracellular vesicles significantly contribute to melanoma progression. Extracellular vesicles include vesicles of various sizes and origins, which can drive cell–cell communication and regulate local and distal organ responses.^79^ In melanoma, small extracellular vesicles can affect distant organs and exacerbate disease spread. Proteomic analysis of small extracellular vesicles secreted by normal skin melanocytes and melanoma cells revealed that the contents of oxidative metabolism‐ and pigmentation‐related proteins in the extracellular vesicles secreted by melanocytes were significantly increased. In comparison, the expression of melanoma antigens and EMT‐related molecules was upregulated in the small extracellular vesicles secreted by melanoma cells.^61^

The current research also provides ideas for understanding the relationship between melanoma with different genetic backgrounds and EVs. On the one hand, EVs have been proven to be efficient carriers of tumour‐specific mutant DNA. Researchers detected mutations in multiple driver genes, including *BRAF* and *NRAS*, in EVs from metastatic melanoma tissue. And their mutation frequency was significantly higher than that of free total DNA in plasma during the same period, reflecting the potential of EVs as high signal intensity biopsy targets.^80^ This advantage also applies in the circulatory system. The use of affinity peptide capture and other methods to isolate EV‐DNA from patient plasma can more sensitively track *BRAF* V600E mutations than traditional free DNA detection, providing a new approach for targeted therapy monitoring.^81^ *BRAF* V600E mutations can be identified in EVs from postoperative lymphatic drainage fluid, correlating with recurrence risk in patients, thereby highlighting the utility of EVs in detecting minimal residual disease.^82^ On the other hand, EVs with different *BRAF* mutation states have some commonalities in regulating core immune escape mechanisms. A comparative study demonstrated that exosomes from both *BRAF* wild‐type and *BRAF* V600E mutant melanoma cell lines modulate the suppression of CD8^+^ T cells via PD‐L1 and IL‐10 signalling pathways.^83^ In short, the nucleic acid composition of EVs can accurately reflect the genetic mutation profile of parental cells, making them superior biopsy materials for efficacy monitoring. However, certain key immunosuppressive functions may have common pathways among different genetic subtypes.

### 3.4 Validation of EV‐related genes using knockout models

Researchers used gene knockout strategies in vitro and in vivo to elucidate the functional roles of key molecules in EV‐mediated melanoma progression. These methods can analyze the relationship between gene expression and EV‐related phenotypes, including biogenesis, content sorting and immune regulation.^84^ Through gene knockout studies, researchers have identified key regulatory factors associated with EV production. For example, knocking out *Rab27A* (a key GTPase that controls the docking of multi‐vesicular vesicles with the plasma membrane) in a cell model significantly reduced the secretion of exosomes and weakened the growth and metastasis ability of melanoma.^85^ Similarly, the absence of *nSMase2* (which drives the formation of ceramide‐dependent exosomes) alters the expression profile of miRNAs in EVs and reduces their metastatic potential. These studies confirm the crucial role of *Rab27A* and *nSMase2* in EV‐mediated intercellular communication.^86^ In addition, the selective loading of molecular cargo into EVs is also regulated by specific sorting mechanisms. Knocking out the RNA‐binding protein hnRNPA2B1 can affect the integration of specific miRNAs into melanoma‐derived EVs, reducing their immunosuppressive ability.^87^ Similarly, knocking out the core component Ago2 of RNA‐induced silencing complexes impairs the sorting of oncogenic miRNAs towards EVs and weakens their tumorigenic effects on receptor cells.^76^ In summary, these gene knockout‐related studies have elucidated the regulation of individual genes on EV function, and the inhibition of EV biogenic regulatory factors such as *Rab27A* or *nSMase2* may provide a theoretical basis for overcoming melanoma.

## 4 BIOMOLECULES IN MELANOMA‐DERIVED EVS

**FIGURE 2:**
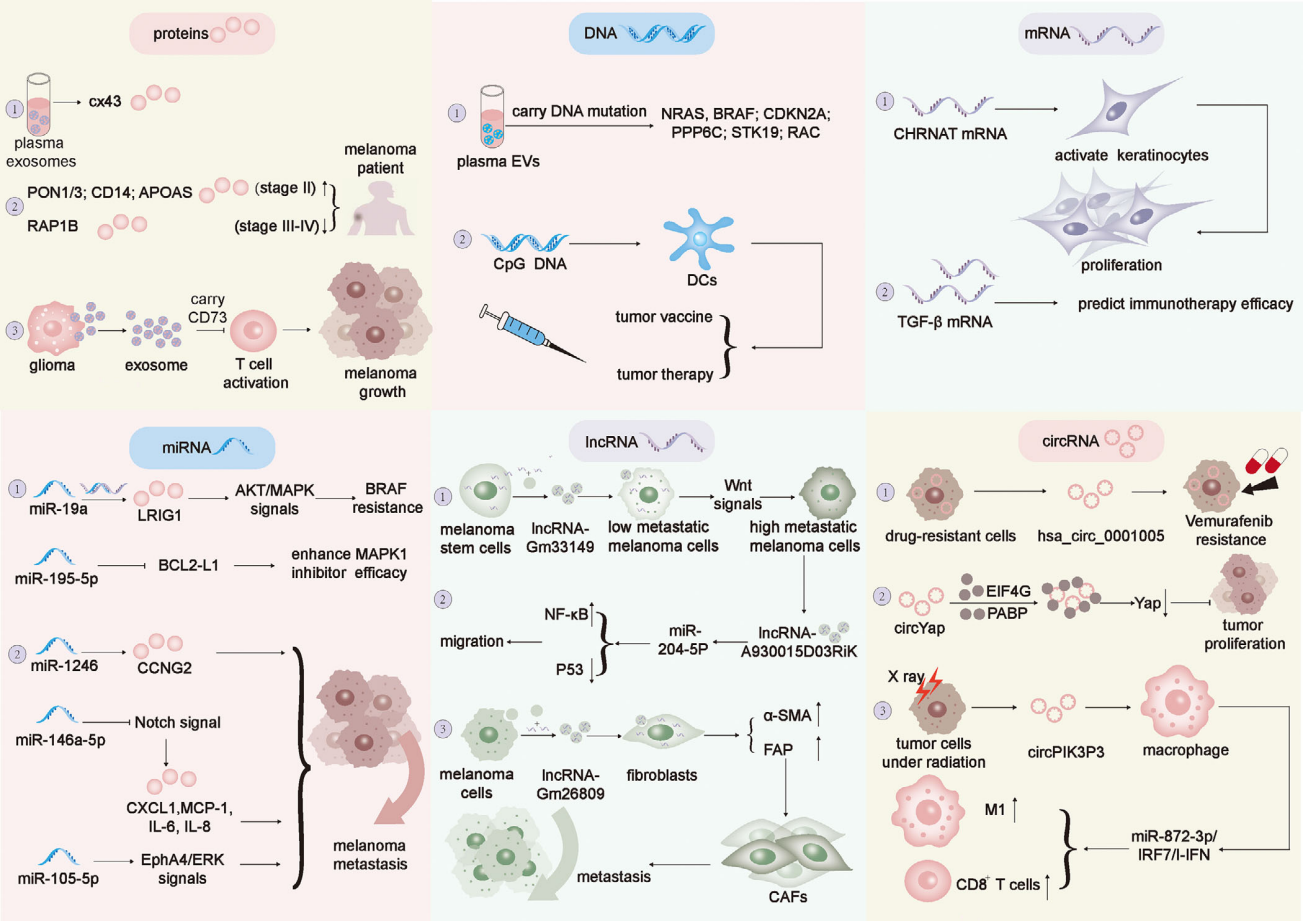
Biomolecules in melanoma‐derived EVs and functional implications. Extracellular vesicles (EVs) derived from melanoma cells carry a diverse range of bioactive molecules originating from their parent cells. Protein components (such as PON1/3, CD14, APOA5 and RAP1B) are derived from melanoma cells, and can serve as markers of disease progression, while melanoma‐associated antigens and metastasis‐related proteins directly participate in immune regulation and metastatic processes. Moreover, the DNA carried by EVs (such as *NRAS*, *BRAF* and other gene mutations) provides the basis for liquid biopsy, and their CpG DNA can also activate DCs. The mRNA carried by melanoma EVs (such as CHRNA7 and TGF‐β mRNA) can be transferred to recipient cells and predict immune therapy response. Importantly, various non‐coding RNAs form a complex regulatory network. MiR‐19a induces BRAF inhibitor resistance by targeting *LRIG1*, while miR‐1246 enhances invasive ability by downregulating CCNG2. LncRNA‐Gm33149 activates Wnt signalling by adsorbing miR‐5623‐3p to promote metastasis. These molecules provide the ability of EVs to reshape the TME and drive malignant processes in melanoma.

Extracellular vesicles, abundant in biomolecules such as proteins, DNA, mRNAs and non‐coding RNAs, are crucial for intercellular communication and cancer progression regulation^88^ (Figure 2). The uptake of extracellular vesicles by different cells and the delivery of these biomolecules to target cells are considered important factors in the influence of extracellular vesicles on the occurrence and development of melanoma^89^ (Table 1).

**TABLE 1:** The function of biomolecules in melanoma‐derived EVs.

Category	Biomolecules	Expression	Function	References
Protein	Cx43	Upregulated	Positively correlated with DFS and OS	^91^
	MAGEA	‐	Function in cancer treatment	^7^
	EMILIN‐1	Upregulated	Closely related to lymph node metastasis	^92^
	PON1/3, CD14 and APOA5	Upregulated	Related to stage II patients	^95^
	RAP1B	Downregulated	Related to stage III‐IV patients	^95^
	CD73	Upregulated	Inhibit T cell proliferation	^96^
DNA	NRAS, BRAF, CDKN2A, PPP6C, STK19 and RAC mutations	Upregulated	Become biomarkers	^80^
	CpG DNA	‐	Used for tumour immunotherapy	^102^
mRNA	α7‐nAChR mRNA	Upregulated	Activate the growth of keratinocytes	^108^
	TGF‐β mRNA	‐	Related to the efficacy of immunotherapy	^109^

### 4.1 Proteins in melanoma‐derived EVs

Proteins are crucial biomolecular components within extracellular vesicles (EVs) derived from melanoma, and they participate in tumour progression, immune regulation and distant metastasis through various mechanisms.^90^ Researchers compared the expression of Cx43 in plasma EVs of 50 healthy individuals and 112 melanoma patients. The results showed that the expression of Cx43 in plasma EVs of melanoma patients was significantly reduced and positively correlated with disease‐free survival and overall survival.^91^ Moreover, MAGEA protein is a melanoma‐associated antigen that plays an important role in cancer progression. Extracellular vesicles can be loaded with MAGEA protein in vivo or loaded with chelating agents in vitro, which ensures the stability of MAGEA protein and its function in cancer treatment.^7^ Multiple studies have shown that melanoma extracellular vesicles are closely related to lymph node metastasis during tumorigenesis. Among them, EMILIN‐1 protein is reduced in expression by protein hydrolysis in extracellular vesicles, which promotes the lymph node metastasis of melanoma. Reducing the level of EMILIN‐1 in extracellular vesicles can inhibit the activity of melanoma and slow down the progression of tumour metastasis.^92^

The protein composition of extracellular vesicles in plasma can reflect the disease progression of melanoma.^93^, ^94^ Through proteomic analysis of plasma extracellular vesicle samples from different stages of melanoma, it was found that PON1/3, CD14 and APOA5 were upregulated in the extracellular vesicles of stage II patients compared with normal individuals, while RAP1B was significantly reduced in the plasma extracellular vesicles of stage III–IV patients.^95^ These changes in the protein profile provide a potential molecular basis for disease staging. On the other hand, CD73 protein is highly expressed in peripheral blood exosomes of glioma patients, and the highly expressed CD73 on the surface of exosomes can be delivered to T cells, subsequently inhibiting T cell proliferation and promoting melanoma growth.^96^ These findings reveal the direct role of vesicular proteins in mediating an immunosuppressive microenvironment.

### 4.2 DNA in melanoma‐derived EVs

DNA carried by extracellular vesicles has emerged as a significant component in the molecular cargo.^97^ The DNA content within EVs, which often reflects the mutational landscape of the tumour, provides a valuable source for liquid biopsy and understanding therapy‐related responses.^98^ Circulating tumour DNA has also received widespread attention and in‐depth research. Currently, circulating tumour DNA obtained from plasma has been found to have specific mutation characteristics, and extracellular vesicles in tumours may also carry specific tumour‐related mutations.^99^, ^100^ By comparing the DNA mutation information of extracellular vesicles obtained from six types of metastatic melanoma tissues with that of tumour tissue DNA, mutations were detected in six genes: *NRAS*, *BRAF*, *CDKN2A*, *PPP6C*, *STK19* and *RAC*. The DNA‐specific mutations detected in these extracellular vesicles also support the possibility of tumour‐derived extracellular vesicles becoming biomarkers.^80^ In addition to reflecting tumour genetics, EV‐DNA can also be involved in therapy‐induced responses. The use of chemotherapy drugs such as irinotecan in cancer patients usually causes the release of extracellular vesicle DNA from the intestine, which is recognized by immune cells and stimulates AIM2 signal transduction and mucosal inflammation.^101^ Furthermore, the immunostimulatory potential of EV‐associated DNA is being explored for therapeutic purposes. Numerous studies have shown that CpG DNA and other substances can activate the body's innate immune response and exert potential anti‐tumour and tumour vaccine effects. CpG DNA on the surface of extracellular vesicles secreted by tumour cells is modified, and its delivery function relies on its uptake by dendritic cells, thereby stimulating immune responses and using for tumour immunotherapy.^102^

### 4.3 mRNAs in melanoma‐derived EVs

mRNA (messenger RNA) has received widespread attention in recent years and has become an effective therapeutic molecule with broad clinical prospects.^103^ In melanoma, extracellular vesicles (EVs) serve as critical vehicles for the intercellular transfer of functional mRNAs, thereby influencing tumour progression and treatment response.^104^ Melanoma cells not only produce histone‐modified extracellular vesicles, but also undergo mRNA transport. During mRNA transmission, three RNA‐binding proteins (with molecular weights of 38, 45 and 65 kDa) can bind to histone‐modified mRNAs in melanoma cells.^105^

In order to avoid degradation of mRNA cargo and reduce immunogenicity, mRNA needs to be delivered using stable, safe and effective delivery systems to achieve therapeutic effects.^106^, ^107^ By stably expressing target mRNAs in extracellular vesicles and fusing them with CD63, researchers have established an efficient strategy for mRNA loading and delivery. The EV‐mRNA delivery system has demonstrated excellent therapeutic effects in a mouse model of metastatic melanoma.^106^ In addition to their therapeutic potential, EV‐derived mRNAs also play a role in promoting tumour malignancy. Bioinformatics analysis found that the expression of the CHRNA7 gene (encodingα7‐nAChR) in patients with highly metastatic melanoma is closely related to poor prognosis. Melanoma extracellular vesicles with metastatic features can activate the growth and migration of keratinocytes by transmitting α7‐nAChR mRNA through the extracellular vesicles.^108^ Furthermore, the mRNA content within circulating EVs shows promise as a predictive biomarker for immunotherapy response. Analysis of the mRNA levels of PD‐L1, TGF‐β and IFN‐γ in plasma EVs of 30 patients with metastatic melanoma after anti‐PD‐1 treatment revealed that melanoma patients with high expression of TGF‐β had longer disease‐free survival (*p* = .006) and overall survival (*p* = .0009). The expression of TGF‐β mRNA in patients’ plasma EVs is closely related to the efficacy of immunotherapy.^109^

### 4.4 miRNAs in melanoma‐derived EVs

MicroRNAs (miRNAs) carried by extracellular vesicles have emerged as pivotal regulators of melanoma progression, influencing processes such as drug resistance, metastasis and immune modulation^110^, ^111^ (Table 2). Recent studies have shown that miRNAs in EVs are involved in the progression of BRAF inhibitor resistance in melanoma and have the potential to become new targets for melanoma treatment. Researchers found that the expression of exosomal miR‐19a was significantly greater in melanoma cells insensitive to BRAF inhibitors than in sensitive cells. Mechanistically, exosomal miR‐19a activates the AKT and MAPK signalling pathways by targeting LRIG1, leading to BRAF inhibitor resistance in melanoma cells.^112^ Extracellular vesicles derived from tumour cells loaded with miRNAs enhance intercellular communication and regulate the TME and cancer progression.^113^ A study on exosomes from highly metastatic and poorly metastatic tumour cells revealed that miR‐1246 loaded in highly metastatic exosomes can enhance the invasive ability of heterogeneous melanoma cells by regulating the expression of *CCNG2*.^114^ Numerous studies have shown that miRNAs secreted by tumour cells in extracellular vesicles can shape a pro‐tumour immune microenvironment. Among them, miR‐146a‐5p is significantly upregulated in extracellular vesicles associated with human melanoma brain metastasis and is transported to astrocytes through extracellular vesicles. Subsequently, the activity of the Notch signalling pathway in astrocytes is inhibited, and the production of oncogenic cytokines (including CXCL1, MCP‐1, IL‐6 and IL‐8) is activated.^115^

**TABLE 2:** Role of non‐coding RNAs in melanoma‐derived EVs.

Category	NcRNAs	Expression	Targets	References
miRNA	miR‐19a	Upregulated	AKT and MAPK signal	^112^
	miR‐1246	Upregulated	CCNG2	^114^
	miR‐146a‐5p	Upregulated	Notch signal	^115^
	miR‐195‐5p	‐	BCL2‐L1	^117^
	miR‐487a‐5p	Upregulated	Nudt21	^118^
	miR‐106b‐5p	Upregulated	EphA4/ERK signal	^119^
lncRNA	lncRNA‐Gm33149	Upregulated	miR‐5623‐3p/Wnt signal	^123^
	LncRNA‐A930015D03Rik	Upregulated	miR‐204‐5p/KLF12	^124^
	LINC01214	Upregulated	miR‐4492/PPP1R11	^126^
	lncRNA‐Gm26809	Upregulated	α‐SMA and FAP	^127^
circRNA	CircRPS5	Downregulated	‐	^134^
	hsa_circ_0001005	Upregulated	miRNAs	^135^
	CircYap	Downregulated	Yap	^136^
	circPIK3R3	Upregulated under radiation therapy	miR‐872‐3p/IRF7 signal	^137^

The expression of specific miRNAs in tumour cells can also modulate the molecular cargo of EVs to enhance drug resistance.^116^ The expression of miR‐195‐5p in tumour cells can increase the expression of inhibitory molecules (including miR‐152‐3p, miR‐195‐5p and miR‐202‐3p) in tumour extracellular vesicles and improve the drug resistance of melanoma. Loading miR‐195‐5p into EVs through electroporation technology can reduce the expression of BCL2‐L1 protein and enhance the efficacy of MAPK inhibitors.^117^ Additionally, dysregulation of certain miRNAs in tumour tissue itself can be transmitted through EVs to stimulate metastasis. Highly metastatic melanoma cells can promote the metastatic characteristics of melanoma cells by directly secreting S100A11/Sec23a protein and using exosomes to deliver miR‐487a‐5p in conjunction with the tumour suppressor *NUDT21*.^118^ Compared with adjacent cancer tissues, the expression of miR‐106b‐5p in melanoma tissues is significantly increased and can be secreted into exosomes to stimulate melanoma metastasis through targeting the EphA4/ERK signalling pathway.^119^

### 4.5 lncRNAs in melanoma‐derived EVs

Long non‐coding RNAs (lncRNAs) present in extracellular vesicles have gained increasing recognition as key regulators in melanoma progression.^120^ These EV‐associated lncRNAs facilitate intercellular communication and contribute to various malignant processes, including metastasis, immune evasion and TME remodelling.^121^, ^122^ LncRNAs in extracellular vesicles affect the mutual communication between cancer stem cells and non‐cancer stem cells during the transmission process. For instance, highly metastatic melanoma stem cells secrete lncRNA‐Gm33149 and encapsulate it in exosomes, which are then taken up by low metastatic melanoma cells. Subsequently, lncRNA‐Gm33149 stimulates the expression of the Wnt signalling pathway by competitively absorbing miR‐5623‐3p, thereby promoting melanoma cells to acquire a strongly metastatic phenotype and exacerbating the malignant progression of cancer.^123^ Similarly, lncRNA‐A930015D03Rik can be secreted into highly metastatic extracellular vesicles and activate the expression of the KLF12 gene by competitively binding to miR‐204‐5p. The subsequent activation of the NF‐κB signalling pathway and downregulation of P53 and the oxidative phosphorylation pathway enhance the invasion and migration characteristics of ordinary melanoma cells.^124^ EV‐derived lncRNAs also play crucial roles in modulating anti‐tumour immunity.^125^ LINC01214 is significantly upregulated in the plasma of melanoma patients and is released into the TME in the form of exosomes, which limits the secretion of TNF‐α, IFN‐γ, perforin and Granzyme‐B by effector T cells as ‘adsorption sponges’ for miR‐4492 and increases the level of PPP1R11.^126^ The exosomes produced by melanoma cells play an important role in stimulating the reprogramming of normal fibroblasts into tumour‐associated fibroblasts. Among them, B16F10 extracellular vesicles deliver and release lncRNA‐Gm26809 to act on fibroblasts, activating the expression of tumour‐associated fibroblast marker genes such as α‐SMA and FAP, thereby promoting the proliferation and metastasis of melanoma cells.^127^

### 4.6 circRNAs in melanoma‐derived EVs

Circular RNA (circRNA) is a type of non‐coding RNA formed by reverse splicing, which is highly conserved.^128^ Unlike linear RNAs, circRNAs, due to their circular closed structure, can tolerate the action of endonucleases and exist in cells for a longer period of time.^129^ In recent years, research has revealed that circRNAs are closely related to various human diseases, including metabolic disorders and cancer. In the process of tumours, circRNAs can serve as biomarkers for diagnosis and prognosis prediction, as well as new targets for tumour treatment.^130^ ^131^ However, the functions and mechanisms of circRNAs in melanoma are still unclear. Numerous studies have shown that many proteins can regulate the biogenesis of circRNAs. For example, HNRNPM is a growth regulator of prostate cancer that can bind to regions containing GU in introns to regulate the biosynthesis of circRNAs and ensure the accuracy of sequence splicing.^132^

Recent studies have found that circRNAs can be secreted into exosomes and exert their functions.^133^ CircRPS5 detected in exosomes can regulate the proliferation, migration, invasion and apoptosis of melanoma cells, and induce cell cycle arrest in vitro, thereby inhibiting the occurrence and development of melanoma and potentially becoming a new therapeutic target for melanoma.^134^ In melanoma, hsa_circ_0001005 is mainly secreted into exosomes by drug‐resistant cells, where it activates resistance‐related signalling pathways and promotes the progression of vemurafenib resistance by competitively binding to four miRNAs. Hsa_circ_0001005 in exosomes is a key molecule that affects the drug resistance of melanoma and provides a new treatment option for melanoma patients.^135^ Moreover, CircYap forms a complex by directly binding to Yap mRNA, eIF4G and PABP, inhibiting the translation initiation of Yap protein and reducing the proliferation, migration and colony formation of tumour cells. It may become a new molecule in regulating Yap expression and a new tool for cancer treatment.^136^ Furthermore, exosomal circRNAs can modulate the tumour immune microenvironment. The exosomal circPIK3R3 secreted by tumour cells is upregulated under radiation therapy stimulation and can be delivered to macrophages, which increases the number of M1 macrophages and promotes the production of I‐IFNs by targeting the miR‐872‐3p/IRF7 signalling pathway. Subsequently, the increased I‐IFN activates the JAK/STAT signalling pathway and the emergence of CD8^+^ T cells, thereby enhancing anti‐tumour immune response in melanoma.^137^

### 4.7 Metabolites and metabolic enzymes in melanoma‐derived EVs

Metabolites and metabolic enzymes carried within EVs have increasingly drawn attention for their ability to reflect parental cell states, influence the TME and modulate responses to drug therapies.^138^ Recent studies have highlighted the diverse metabolic components in EVs and their crucial role in melanoma progression. Metabolites within plant‐derived EVs exhibit tumour progression‐modulating functions. For instance, EVs extracted from citrus fruits possess a unique metabolic profile characterized by abundant amino acids, organic acids and lipids, distinctly differing from the high‐sugar components found in fruit juices. Plant‐derived EVs can inhibit melanoma growth by causing cell cycle arrest, modulating cyclin expression and suppressing the Akt/ERK signalling pathway, underscoring the therapeutic potential of natural EVs and their metabolites in cancer treatment.^139^ Furthermore, multi‐omics analysis of EVs in melanoma patients’ blood directly elucidates the role of EVs metabolites in melanoma progression. Through the joint analysis of proteomics and metabolomics, it was found that the expression levels of lipid metabolites (such as lyso PC a C18:2 and PC ae C44:3) and proteins (such as APOC4 and PRG4) in patients’ plasma EVs can indicate varying disease progression.^140^ This provides new biomarkers and targets for early detection, prognostic assessment and personalized treatment of melanoma. Based on a deep understanding of the metabolic function of EVs, targeting metabolic pathways has become a new approach to enhance anti‐tumour efficacy. Engineering EVs loaded with doxorubicin and the glycolysis inhibitor GSK2837808A enables direct tumour cell killing, induces immunogenic cell death and reverses the immunosuppressive effects of glycolysis, thereby effectively intervening in tumour cell metabolism. The combination of immune checkpoint inhibitors (anti‐CTLA‐4) led to the eradication of mouse melanoma and the establishment of durable immune memory.^42^ These studies reveal the central role of EV‐mediated metabolic reprogramming in melanoma progression.

## 5 EVs IN THE TME

TME is a complex and dynamic ecosystem composed of tumour cells and various non‐tumour cells, including immune cells and stromal cells.^141^ The extracellular vesicles secreted by these diverse cellular components serve as crucial mediators of intercellular communication, actively modulating tumour progression, immune responses and therapeutic outcomes^142^ (Table 3).

**TABLE 3:** The function of EVs among various cells in the TME.

Cell type	Biomolecules	Parent cell	Receptor cell	Function	References
Epithelial cell	circRNA102927	Melanoma cell	Epithelial cell	Affect the EMT	^144^
	OX40L, CD80 and PD‐L1	Epithelial cell	T cell	Enhance the anti‐tumour effect	^145^
Stromal cell	HSP90/p‐IKKα/β	Melanoma cell	Stromal cell	Promote angiogenesis	^148^
	DR5‐scFv	NK cell	Stromal cell	Prolong mouse survival	^149^
	CD9	Tumour‐associated fibroblast	‐	Inhibit the growth of melanoma	^151^, ^152^
	miR‐92b‐3p	Melanoma cell	Stromal cell	Reduce PTEN levels and CAFs‐related gene expression	^49^
	miR‐214	Stromal cell	Stromal cell	Regulate TME and promote tumour growth	^153^
Endothelial cell	miR‐1246	Metastatic melanoma cell	Endothelial cell	Increase endothelial cell resistance to 5‐FU	^156^
	NGFR and p75NTR	Melanoma cell	Lymphatic endothelial cell	Promote angiogenesis	^158^
	Tumour antigens	Melanoma cell	Lymphatic endothelial cell	Induce a reduction in specific CD8^+^ T cells through MHC‐I	^160^
Cancer stem cell	mir100Hg	Melanoma stem cell	Melanoma cell	Affect the expression of glycolysis‐related genes	^164^
	miR‐592	Melanoma stem cell	Melanoma cell	Activate the PTPN7/MAPK signalling axis	^165^
	miR‐4535 and miR‐1268a	Low metastatic melanoma	High metastatic melanoma	Regulate autophagy‐related pathways	^166^
T cell	PD‐L1	Melanoma cell	T cell	Activate CREB protein and STAT signalling pathway	^48^
	miR‐25‐3p, miR‐215‐5p, miR‐375	CD4^+^ T cells	CD8^+^ T cells	Participate in the occurrence and development of melanoma	^170^
	Mel TregS	Treg cells	‐	Clinical monitoring of melanoma	^172^
Macrophage	miR‐1246	Melanoma cell	Macrophage	Promote tumour metastasis	^178^
	CD36	Melanoma cell	Macrophage	Activate the production of M2 macrophages	^60^
	miR‐214	Melanoma cell	Macrophage	Affect endothelial cell permeability	^181^
	RAB27A	Tumour‐associated macrophage	CD8^+^ T cell	Play an important role in immune regulation	^184^
MDSC	let‐7e‐5p, miR‐125a‐5p and miR‐125b‐5p	Melanoma cell	Normal myeloid cell	Lead to their transformation into MDSC‐like cells	^188^
	VEGF and osteopontin	Melanoma cell	‐	Induce an increase in B cells and myeloid suppressor cells	^189^
NK cell	ALIX and CD63	NK cell	Melanoma cell	Achieve tumour‐killing function	^193^
	Hsp70	Melanoma cell	NK cell	Kill melanoma cells	^197^
Neutrophil	EVs	High metastatic melanoma	Neutrophil	Stimulate neutrophil extracellular trap formation and N2‐type neutrophil	^199^
Microbiota	EVs	Photosynthetic cyanobacteria	Melanoma cell	Hinder melanoma recurrence and migration	^200^
	Bacterial outer membrane vesicles	*E. coli*	‐	Enhance synergistic anti‐tumour effects	^203^

### 5.1 EVs and epithelial cells

Extracellular vesicles mediate crucial interactions involving epithelial cells within the melanoma TME, influencing processes such as the EMT and serving as platforms for engineered immunotherapies.^143^ The biomolecules delivered by exosomes play important roles in the EMT progression of melanoma. Using high‐throughput sequencing technology to detect gene expression in primary and metastatic melanoma exosomes, 12 differentially expressed circRNAs were screened. Among them, circRNA102927 in melanoma exosomes can promote the progression of melanoma by affecting the EMT.^144^

Extracellular vesicles derived from epithelial cells have been proven to be safe drug delivery systems in multiple studies and can induce immune responses in the body through protein modification. Researchers have designed extracellular vesicles carrying OX40L, CD80 and PD‐L1 immune regulatory proteins, which effectively activate human and mouse T cell responses and alleviate disease progression. Moreover, the combination of engineered extracellular vesicles and anti‐CTLA‐4 also effectively enhances the anti‐tumoUr effect in melanoma mice.^145^

### 5.2 EVs and stromal cells

Stromal cells are one of the most important cellular components in the TME in melanoma.^146^ The exosomes secreted by melanoma cells can act on cancer‐related fibroblasts and activate pro‐tumour responses by stimulating chemokines and cytokines.^147^ In‐depth research has shown that extracellular vesicles derived from melanoma cells can encapsulate HSP90/p‐IKKα/β protein complexes and transport them into cancer‐associated fibroblasts (CAFs), which promote angiogenesis and cancer progression by activating the NF‐κB/CXCL1 signalling pathway.^148^ Exosomes derived from melanoma cells are rich in miR‐92b‐3p, which is involved in the transition of fibroblasts to CAFs. MiR‐92b‐3p can be transported by exosomes and taken up by normal fibroblasts, promoting melanoma cell migration by reducing PTEN levels and inducing CAF‐related gene expression.^49^ A few EVs or engineered EVs can inhibit the growth and migration of melanoma by acting on immune suppressive stromal cells in the TME. Researchers have designed a PDGFR transmembrane domain to deliver DR5‐single‐chain variable fragments (DR5‐scFv) to the surface of extracellular vesicles, which can rapidly induce apoptosis in CAFs, MDSCs and DR5^+^ tumour cells, significantly slowing down the progression of DR5^+^ melanoma and prolonging mouse survival.^149^ Extracellular vesicles derived from fibroblasts can also affect the progression of melanoma.^150^ Researchers isolated extracellular vesicles from elderly and young fibroblasts and reported no difference in size or morphology, but the proteins in the extracellular vesicles underwent significant changes. Among them, the expression of CD9 in the extracellular vesicles of elderly fibroblasts is significantly reduced, which promotes an increase in the angiopoietin‐like protein ANGPTL2 and exacerbates angiogenesis in melanoma.^151^ In addition, CD9‐positive extracellular vesicles secreted by tumour‐associated fibroblasts have a longer 5‐year survival period than those secreted by CD9‐negative extracellular vesicles. And CD9‐positive CAF‐derived extracellular vesicles can significantly inhibit the growth of melanoma.^152^ Moreover, miR‐214 is expressed mainly in the stromal cells of tumour samples and can be delivered to melanoma cells via exosomes to regulate the TME and promote tumour growth and proliferation.^153^

### 5.3 EVs and endothelial cells

The effects of extracellular vesicles on endothelial cells are gradually being explained.^154^ Researchers have isolated extracellular vesicles from four different types of melanoma cells and added them to the supernatant of endothelial cells. The results of in vitro experiments showed that the expression of the angiogenesis‐related αvβ5/VEGF pathway, rather than the αvβ3/TNF‐α pathway, was significantly altered in endothelial cells, leading to enhanced migration and tube formation abilities of endothelial cells.^155^

Tumour endothelial cells are involved in the important process of cancer metastasis. MiR‐1246 is significantly upregulated in the extracellular vesicles of metastatic melanoma cells and can be delivered to tumour endothelial cells, affecting endothelial cell adhesion function and regulating endothelial cell barrier construction. Mechanistically, miR‐1246 in EVs promotes the lung metastasis of melanoma by activating the expression of STAT3, ICAM‐1 and E‐cadherin in endothelial cells.^156^ In addition, tumour extracellular vesicles with high metastatic potential can encapsulate miR‐1246 and be taken up by endothelial cells, which activate the STAT3 and Akt signalling pathways and increase endothelial cell resistance to 5‐FU.^157^ The extracellular vesicles produced by melanoma cells can transmit information to endothelial cells and reshape the pre‐metastatic niche of lymph nodes. Extracellular vesicles isolated from melanoma cells are rich in growth factor receptors such as NGFR and p75NTR, which are absorbed by lymphatic endothelial cells and promote angiogenesis and tumour cell adhesion by increasing the expression of ICAM‐1, NF‐κB and ERK kinases in endothelial cells.^158^, ^159^ Additionally, Noelle Leary et al.^160^ found that extracellular vesicles secreted by melanoma cells can act specifically on lymphatic endothelial cells and induce changes in gene expression and cell proliferation. Meanwhile, the extracellular vesicles of melanoma cells deliver tumour antigens to lymphatic endothelial cells and reduce the number of specific CD8^+^ T cells through MHC‐I cross‐presentation.

### 5.4 EVs and cancer stem cells

Melanoma is highly invasive and poses a serious threat to the lives of patients. Melanoma cancer stem cells have self‐renewal ability, which is an important factor leading to metastasis and chemoresistance.^161^ Compared with those of melanoma cells, there are significant differences in the metabolomic characteristics of exosomes secreted by melanoma cancer stem cells.^162^

The bioactive components in extracellular vesicles mediate the communication between cancer stem cells and tumour cells, and regulate the malignant progression of melanoma.^163^ The expression of lncRNA‐mir100Hg is significantly upregulated in melanoma stem cells and is transported into tumour cells through exosomes. Subsequently, Mir100Hg competitively binds to endogenous miR‐23a‐3p and miR‐16‐5p to affect the expression of glycolysis‐related genes and promote the metastasis of melanoma cells.^164^ An exploration of the interaction between cancer stem cells and tumour cells revealed that miR‐592 mediated the mutual communication between melanoma stem cells and melanoma parent cells. Extracellular vesicles derived from melanoma stem cells deliver miR‐592 to melanoma parent cells, which then activate the PTPN7/MAPK signalling axis and enhance the metastatic ability of melanoma parent cells.^165^ In addition, another study found that miR‐4535 and miR‐1268a in exosomes mediate the transition from low‐metastatic melanoma to high‐metastatic melanoma by regulating autophagy‐related pathways.^166^

### 5.5 EVs and T cells

Extracellular vesicles secreted by tumour cells play crucial roles in activating effector T cells during the immune process.^167^ Exosomes derived from melanoma can carry various tumour antigens and activate the maturation of dendritic cells, subsequently inducing effector CD8^+^ T cells to combat tumour growth and migration.^168^ The PD‐L1 molecules derived from tumour cell‐derived exosomes can activate CREB protein and STAT signalling pathway, which enhances T cell lipid metabolism in humans and mice and exacerbates T cell ageing. The use of drugs to inhibit the production of EVs derived from tumour cells promotes the anti‐tumour effects of adoptive T cell therapy and anti‐PD‐L1 immunotherapy.^48^ These studies elucidate the mechanism of the association between tumour‐derived exosomes and cancer progression and the development of immunotherapy resistance.^169^ The latest research shows that extracellular vesicles derived from CD4^+^ T cells can participate in the occurrence and development of melanoma. Extracellular vesicles are secreted by CD4^+^ T cells and can activate the anti‐tumour effect of CD8^+^ T cells by delivering miRNAs (including miR‐25‐3p, miR‐215‐5p, miR‐375, etc.) rather than affecting Tregs (regulatory T cells) changes.^170^

Tregs are cells that exert immunosuppressive effects and are involved in the occurrence and development of melanoma.^171^ The spatial transcriptome sequencing results showed that upregulation of FOXP3 and BATF expression in melanoma T cells led to their polarization towards Tregs. At the same time, large amounts of clinical sample data have revealed the existence of Mel TregS (Treg‐specific regulatory tag set) in melanoma, and the level of Mel TregS in the exosomes of melanoma can be used to distinguish between healthy individuals, early‐stage melanoma patients and late‐stage melanoma patients, which has the potential to become a new strategy for clinical monitoring of melanoma.^172^

In recent years, CAR T cell (chimeric antigen receptor) therapy has shown good results in combating haematological tumours, but its efficacy in solid tumours is very limited.^173^ To improve the efficacy of CAR T therapy in solid tumours such as melanoma, researchers have found that extracellular vesicles secreted by tumour cells can fuse with CAR T cells and limit their application. In a mouse tumour model, inhibiting tumour‐derived extracellular vesicles can significantly enhance the activity and anti‐tumour effect of CAR T cells.^174^

### 5.6 EVs and macrophages

According to the analysis of cytokine profile data, it was found that EVs derived from melanoma cells can increase the expression of tumour‐associated macrophage marker factors, reshape the immunosuppressive microenvironment and support the growth and metastasis of melanoma.^175^ The uptake of extracellular vesicles secreted by tumour cells by macrophages in the TME can shape the pre‐metastatic niche of the tumour.^176^, ^177^ Collect extracellular vesicles from mice inoculated with melanoma and neuroblastoma, and it was found that mouse macrophages can take up extracellular vesicles that transmit miR‐1246 and promote tumour metastasis through in vitro experiments.^178^ Our further research found that patients with metastatic melanoma also have upregulation of similar marker factors in their bodies compared with healthy individuals.^179^ Extracellular vesicles secreted by melanoma cells that do not express PD‐L1 can affect the expression of macrophage‐related factors, including cytokines (TGFβ1, IL6 and IL10) and chemokines (CXCL2, CCL2 and CCL3), which reduce the number of M2 macrophages and slow down tumour growth rate.^180^ Suman et al. evaluated the expression of CD36 in lymphatic‐derived extracellular vesicles in the SKCM database and found that melanoma‐derived extracellular vesicles can upregulate CD36 expression and activate the production of M2 macrophages. At the same time, the addition of melanoma‐derived extracellular vesicles to endothelial cells and monocytes revealed a significant increase in CD36 expression, indicating that melanoma‐derived extracellular vesicles rely on CD36 expression to promote the formation of pre‐metastatic immune niches.^60^ Additionally, the extracellular vesicles secreted by melanoma are rich in miR‐214, which activates macrophages and increases the levels of proinflammatory factors. Subsequently, changes in the TME affect endothelial cell permeability and enhance the intra‐vascular migration of melanoma cells.^181^ Moreover, keratinocytes and fibroblasts can deliver melanoma‐derived vesicles, which are further engulfed by macrophages. Macrophages polarize into a pro‐tumour phenotype, inducing VEGF expression and promoting the shaping of the microenvironment of melanoma metastatic niche^62^, ^182^

Macrophages are reprogrammed into tumour‐associated macrophages (TAMs) in the TME, which can stimulate the production of exosomes and play an important role in immune regulation.^183^ Through analysis of melanoma tissue samples, it was found that tumour‐associated macrophage exosomes can significantly inhibit the appearance of CD8^+^ T cells. Further single‐cell sequencing analysis revealed a negative correlation between the expression of the *RAB27A* gene, which is related to TAMs, and the number of infiltrating CD8^+^ T cells.^184^ To investigate the role of extracellular vesicles secreted by macrophages after treatment with doxorubicin (DOX) in regulating melanoma cell proliferation, researchers treated macrophages with doxorubicin and doxorubicin‐loaded nanoparticles (DOX‐CDNSs) and examined the effect of extracellular vesicles on tumour proliferation. The results showed that nanoparticles loaded with doxorubicin had greater toxic effects on cancer cells, and they could induce macrophages to secrete pro‐cancer extracellular vesicles in the TME.^185^

### 5.7 EVs and MDSCs

Numerous studies have found that the function of myeloid cells is also regulated by extracellular vesicles derived from tumours.^186^ Extracellular vesicles isolated from tumour cells can act on myeloid cells and induce an increase in MDSCs, which then restrict the activation and tumour killing ability of NK cells and T cells.^187^ Lasser et al. investigated the transfer trajectory of exosomal miRNAs derived from melanoma cells in a transgenic mouse melanoma model, and the results showed a significant increase in the expression of let‐7e‐5p, miR‐125a‐5p and miR‐125b‐5p in MDSCs. And adding these miRNAs to normal myeloid cells can activate the NF‐κB signalling pathway, leading to their transformation into MDSC‐like cells.^188^ The imbalance between immature myeloid cells and immune suppressive cells is a key factor leading to the progression of tumour immune escape. A proteomic study found that exosomes derived from melanoma cells are closely related to the expression of angiogenesis‐related proteins, including VEGF and osteopontin, which can lead to hematopoietic dysfunction, decreased bone marrow cells and expansion of myeloid suppressor cells. Further in vitro studies have also found that exosomes secreted by melanoma induce an increase in B cells and myeloid suppressor cells, leading to hematopoietic dysfunction and immune escape.^189^ Due to the immunosuppressive effect of MDSCs, researchers have utilized extracellular vesicles to target MDSCs. By specifically binding the miR‐9 sequence to G4‐CSSD9 and preparing nanocapsules through membrane compression with mesenchymal stem cells, the therapeutic effect on melanoma is enhanced by delivering them to MDSCs.^190^

### 5.8 EVs and NK cells

Natural killer cells can effectively restrict the growth and migration of tumour cells.^191^ The extracellular vesicles produced by NK cells have biological activity and play an anti‐tumour role in both in vivo and in vitro experiments.^192^ The extracellular vesicles secreted by NK cells express two proteins, ALIX and CD63, which regulate the proliferation of melanoma cells, achieve tumour killing functions and reduce the occurrence of side effects by producing tumour necrosis factors.^193^ EVs derived from NK cells have broad therapeutic prospects. The use of factors such as IL‐12 to activate NK‐92 cells and primary NK cells results in the release of more extracellular vesicles, which can penetrate the tumour core and better target solid tumours.^194^ Researchers increased the production of extracellular vesicles by designing a bioreactor for cultivating NK cells and found that the extracellular vesicles produced by NK cells can kill melanoma cells and inhibit the activity of cancer cells in vivo and in vitro.^195^, ^196^ In addition, the increased Hsp70 in melanoma cell line B16 can be encapsulated in extracellular vesicles and penetrate nearby cells for transport to natural killer cells, thereby killing melanoma cells.^197^ Furthermore, injecting exosomes produced by radiation‐irradiated melanoma cells into tumours significantly inhibited tumour growth compared with non‐irradiated exosomes, an effect closely related to increased NK cell infiltration.^80^

### 5.9 EVs and neutrophils

Neutrophils exhibit two phenotypes in tumours: promoting tumour growth and inhibiting tumour growth, which are respectively referred to as TAN‐N2 and TAN‐N1.^198^ Highly metastatic melanoma cells can generate extracellular vesicles and act on neutrophils by regulating the CXCR2 and PI3K/Akt signalling pathways to stimulate neutrophil extracellular trap formation. In addition, highly metastatic melanoma exosomes upregulated the expression of N2‐type neutrophil marker genes, including ARG1, VEGF and CXCR4, thereby reshaping the melanoma immune microenvironment and exacerbating the malignant progression of the tumour.^199^

### 5.10 EVs and the microbiota

The interaction between microbes and their vesicles in the TME presents a novel area of research. *Photosynthetic cyanobacteria* encapsulated in hydrogel can produce O_2_, thereby promoting oxidative stress, inducing tumour cell death and inhibiting tumour recurrence by blocking HIF1α signalling axis. At the same time, the generated O_2_ synergizes with extracellular vesicles derived from *photosynthetic cyanobacteria* to promote wound repair after melanoma resection and hinder melanoma recurrence and migration.^200^

**FIGURE 3:**
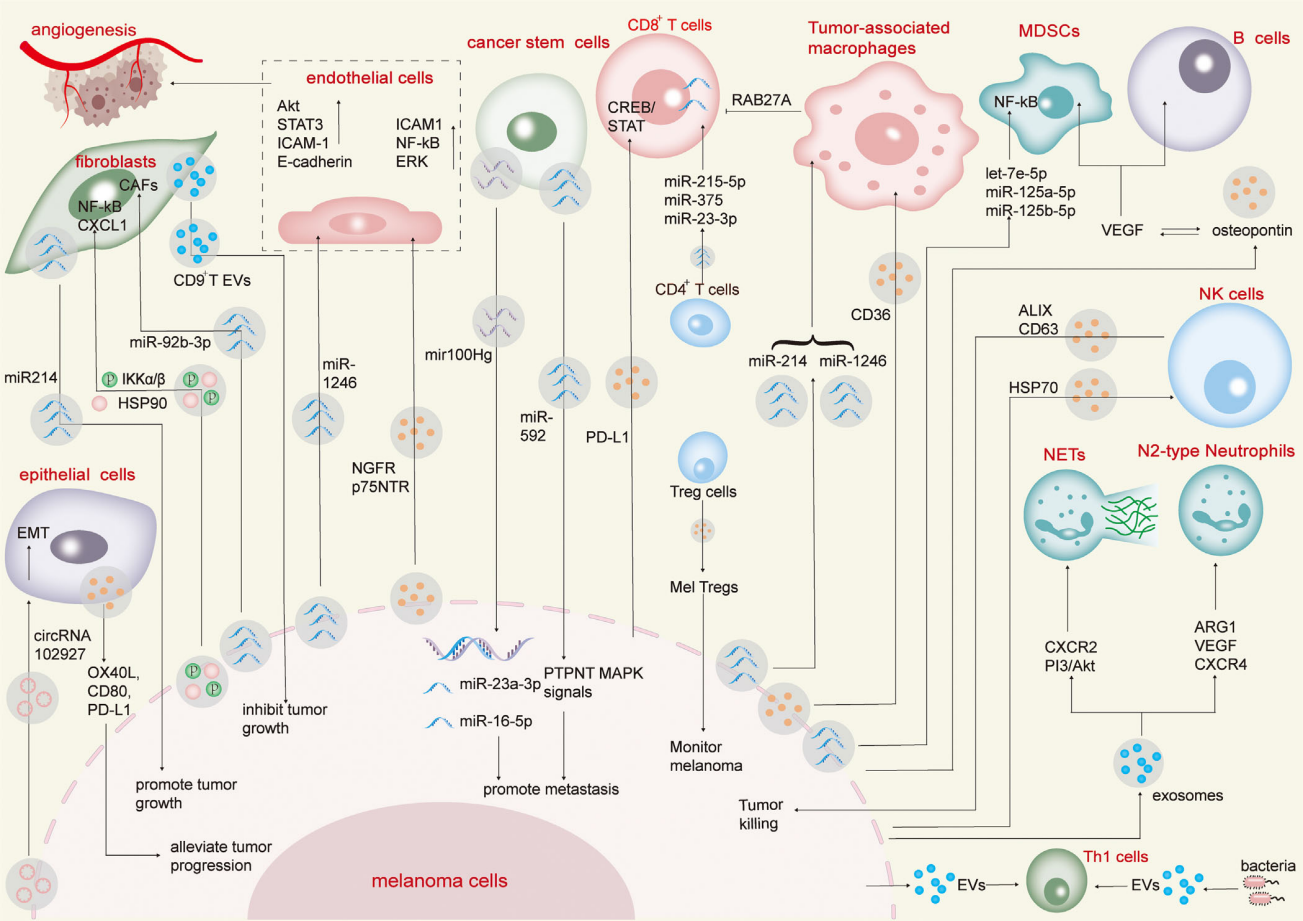
The functions of EVs among various cells in the TME. The complex communication network formed by EVs and various components in the TME is present in melanoma. In terms of immune regulation, melanoma‐derived EVs activate astrocytes by delivering miR‐146a‐5p to promote brain metastasis. EVs derived from CD4^+^ T cells can activate CD8^+^ T cells, while EVs from tumour‐associated macrophages inhibit their infiltration. For stromal cells, melanoma‐derived EVs induce fibroblasts to CAFs transformation and promote angiogenesis by delivering miR‐92b‐3p and HSP90 complexes, but CAFs‐derived CD9^+^ EVs can inhibit tumour growth. When affecting the vascular system, EVs rich in miR‐1246 promote endothelial migration and lung metastasis by activating STAT3 signals, while NGFR/p75NTR^+^ EVs promote lymphangiogenesis by activating the NF‐κB/ERK pathway. In addition, EVs also amplify MDSCs through specific miRNAs and polarize N2‐type neutrophils through the CXCR2/PI3K/Akt pathway to shape an immunosuppressive environment, while EVs derived from NK cells exert anti‐tumour effects. The synergistic effect of multiple cell types in the TME regulates tumour progression, metastasis and immune escape.

Bacterial outer membrane vesicles can activate innate immune responses and have attracted attention as a new strategy for cancer treatment and prevention.^201^ The combination therapy of synthetic bacterial vesicles and melanoma extracellular vesicles can stimulate Th1‐type immune cells and produce balanced antibodies, which leads to the regression of melanoma in tumour‐bearing mice. Meanwhile, the combination of synthetic bacterial vesicles and anti‐PD‐1 inhibitors can synergistically promote the safety and efficacy of immunotherapy.^202^ Since the surface of bacterial outer membrane vesicles can activate anti‐tumour immune effects through antigen presentation, researchers modified specific tumour antigens on the surface of bacterial outer membrane vesicles to enhance synergistic anti‐tumour effects and inhibit the lung metastasis process of melanoma^203^ (Figure 3).

## 6 KEY PATHWAYS OF IMMUNE ESCAPE AND CHEMORESISTANCE MEDIATED BY EVS

### 6.1 Key signalling pathways in EV‐mediated immune escape

Extracellular vesicles derived from melanoma systematically regulate the activity of multiple signalling pathways in receptor cells by delivering specific proteins, nucleic acids and lipid molecules, thereby reshaping the tumour immune microenvironment.^204^ A deep understanding of these signalling networks is important for achieving precise immunotherapy interventions.

The PD‐1/PD‐L1 pathway is the most well‐known and thoroughly researched among immune escape pathways. The surface of exosomes released by melanoma cells carries functional PD‐L1 molecules, and their membrane topology is consistent with the cell surface PD‐L1.^205^, ^206^ The extracellular domain is exposed on the surface of exosomes and can directly bind to the PD‐1 receptor on the surface of T cells. Interferon‐γ can significantly upregulate the expression level of extracellular vesicle PD‐L1 and enhance its binding ability with PD‐1.^207^ In addition, PD‐L1‐positive exosomes suppress CD8^+^ T cell proliferation, cytokine production (IFN‐γ, IL‐2 and TNF‐α) and cytotoxic activity. In vivo experiments demonstrate that melanoma‐derived exosome injections significantly enhance tumour growth, decrease tumour‐infiltrating CD8^+^ T cells and suppress PD‐1^+^ CD8^+^ T cell proliferation in the spleen and lymph nodes.^208^

The NF‐κB pathway is a conserved signalling mechanism activated by danger signals and inflammatory factors. Melanoma‐derived EVs can modulate NF‐κB signalling in immune cells. For example, EVs derived from mouse melanoma cell lines can activate NF‐κB in RAW264.7 macrophages and primary macrophages, altering the release of inflammatory cytokines and chemokines.^209^ EVs from melanoma patients’ plasma activate NF‐κB in their own peripheral blood CD8^+^ T cells, and inhibiting NF‐κB with drugs can counteract the EV‐induced suppression of T cell proliferation. The possible mechanisms by which EV activates NF‐κB include signalling through the S100A8/A9 protein via TLR4.^210^

The TGF‐β/Smad pathway significantly contributes to immune suppression mediated by EVs. Melanoma‐derived exosomes carry transforming growth factor‐β or its related miRNA, which can exert immunosuppressive effects by activating the Smad signalling cascade in receptor cells.^211^ Research has shown that the inhibitory effect of melanoma exosomes on dendritic cell maturation can be reversed by TGF‐β blocking antibodies, suggesting a key role of TGF‐β in this process.^212^, ^213^ Moreover, melanoma‐derived EVs can weaken tumour immunity by triggering apoptosis in immune effector cells. EVs isolated from human melanoma cell lines and melanoma patient serum contain FasL and TRAIL, which can trigger apoptosis in melanoma‐specific CD8^+^ T cells derived from patients. Blocking antibodies against FasL can partially mitigate EV‐induced apoptosis in T cells.^214^

### 6.2 Key signalling pathways in EVs‐mediated chemoresistance

The MAPK/ERK pathway is one of the core mechanisms of BRAF/MEK inhibitor resistance. The MAPK signalling pathway is crucial for melanoma proliferation, with around 50% of patients having oncogenic *BRAF* mutations.^215^ Melanoma EVs reactivate MAPK/ERK signalling in recipient tumour cells by delivering specific miRNAs (such as miR‐19a) or receptor proteins.^112^ Research has shown that even in the presence of BRAF/MEK inhibitors, EV‐mediated paracrine signals can still maintain the activation of downstream pro‐survival signals, which is one of the core mechanisms of acquired resistance. Meanwhile, MAPK inhibitors modulate the expression of NKG2D and other NK activation ligands in both *BRAF* mutant and wild‐type melanoma cells, indicating the immune system's involvement in the emergence of resistance to targeted therapies.^216^

Additionally, the PI3K/Akt/mTOR pathway is a core regulator of cell survival, proliferation and metabolism.^217^ Melanoma cells with acquired resistance to vemurafenib exhibit a marked increase in the activation of the survival protein Akt. The active ingredients transmitted by EVs can help tumour cells avoid apoptosis induced by targeted therapy or chemotherapy by activating the PI3K/Akt/mTOR signalling. The activation of this pathway is associated with resistance to various treatments, including BRAF inhibitors, chemotherapy and radiotherapy. The significant interaction between the PI3K/Akt and MAPK pathways plays a crucial role in sustaining the survival of drug‐resistant cells through their combined activation.^218^ Moreover, abnormal activation of the Wnt/β‐catenin pathway not only participates in immune escape but also mediates targeted therapy resistance by promoting stemness phenotype. Research has shown that highly metastatic melanoma cells utilize exosomal miRNAs to activate the Wnt/β‐catenin signalling pathway, creating a positive feedback loop that enhances metastasis and treatment resistance.^219^ Furthermore, the NF‐κB pathway is crucial in mediating resistance to BRAF inhibitors. EVs can promote NF‐κB activation by transmitting relevant factors, ultimately driving the acquired resistance phenotype.^220^

The above signal pathways do not operate in isolation, but form a complex cross‐dialogue network. For instance, Akt activation can phosphorylate and stabilize β‐catenin, connecting survival signals with proliferative signals. STAT3 and NF‐κB signals can synergistically amplify immunosuppressive effects. EVs can simultaneously deliver goods from multiple pathways (such as specific miRNAs and multiple kinase proteins) to receptor cells, thereby efficiently and synergistically driving immune escape and therapeutic resistance phenotypes.

## 7 EVS IN CLINICAL APPLICATION

### 7.1 EVs as diagnostic biomarkers

**FIGURE 4:**
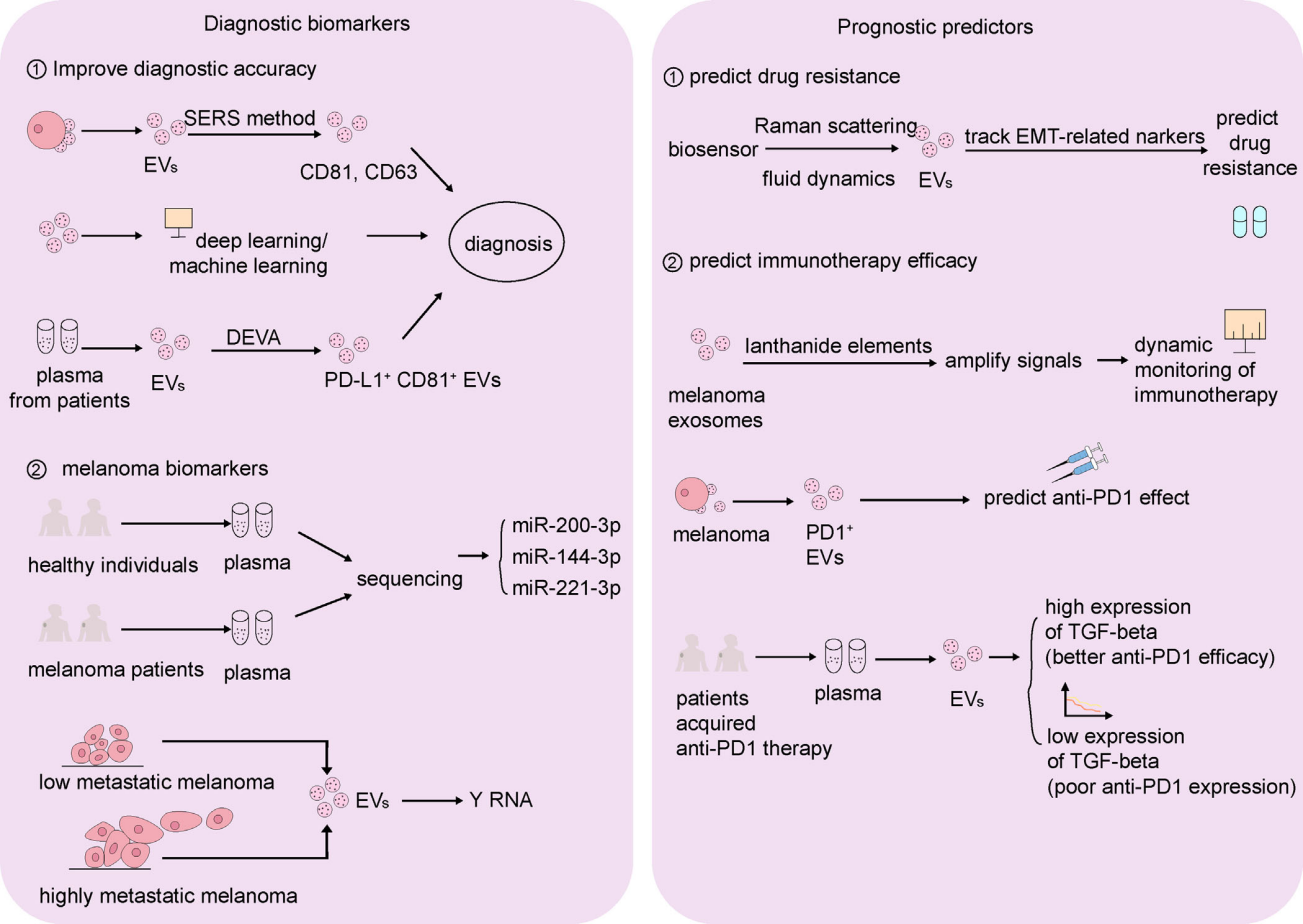
EVs as diagnostic biomarkers and prognostic predictors in melanoma. The strategy of using EVs as biomarkers for melanoma mainly includes three applications: diagnosis, prognosis prediction and treatment monitoring. In terms of diagnosis, various techniques have been developed to improve sensitivity and specificity, such as using surface‐enhanced Raman spectroscopy (SERS) to detect EV protein markers, quantifying PD‐L1^+^ CD81^+^ EVs in plasma through droplet analysis (DEVA), identifying miRNA combinations that can distinguish patients from healthy individuals and distinguishing metastatic and non‐metastatic diseases through Y RNA expression levels. In prognosis and prediction, the combination of machine learning and Raman spectroscopy can improve diagnostic accuracy. In addition, EVs can be used to track epithelial–mesenchymal transition (EMT) markers to predict drug resistance, and lanthanide‐based signal amplification technology can monitor EV dynamics during immunotherapy.

Melanoma has strong invasion and migration ability, and is an important factor in the death of skin tumour patients.^221^ To achieve early diagnosis and treatment, extracellular vesicles present in liquid biopsies have emerged as promising minimally invasive biomarkers for tumours^222^ (Figure 4). Researchers used the SERS method (unlabelled surface‐enhanced Raman spectroscopy) combined with micro‐plasma co‐culture to detect molecular markers of melanoma‐derived exosomes. The results showed that extracellular vesicle marker proteins (including CD81 and CD63) exhibited Raman shifts within 943–1030 and 1304–1561 cm^−1^, which were different from the 1394/1404, 1271 and 1592 cm^−1^ ranges of tumour cells. This provides a promising strategy for the clinical diagnosis of melanoma extracellular vesicles.^223^ At present, precise quantification of PD‐L1^+^ EVs in clinical samples still has significant research value. A study has developed a new extracellular vesicle analysis method DEVA (droplet‐based extracellular vesicle analysis) that can sensitively detect the content of double positive exosomes expressing PD‐L1 and CD81 in the plasma, which has great application value in clinical diagnostic guidance.^224^

Conventional diagnosis usually relies on traditional morphological assessments such as clinical visual examination, dermatoscopy and histopathological biopsy.^225^ Compared with the limitations of traditional imaging in detecting micro‐metastases, EV biomarkers provide early signals at the molecular level. In a study involving 92 participants, certain miRNA combinations in plasma exosomes, including miR‐200c‐3p, miR‐144‐3p and miR‐221‐3p, successfully differentiated patients from healthy individuals and distinguished melanoma from benign nevi.^226^ In addition, sequencing research results revealed that Y RNA in metastatic oral melanoma samples (including tissues, blood and blood‐derived exosomes) was significantly reduced compared with non‐metastatic melanoma. Plasma exosomal Y RNA (AUC area of  .981, *p*‐value of  .0002) has the potential to become a biomarker for diagnosing metastatic melanoma, but it is necessary to increase the sample size to explore its reliability as a clinical biomarker.^227^ The micro‐needle patch (ExoPatch) that captures EVs in the interstitial fluid of the skin can effectively distinguish cancerous and healthy tissues in preclinical models by detecting melanoma‐specific antigens (MCAM/MCSP) on EVs.^228^ However, further exploration of the differences between novel biomarkers and common ctDNA biomarkers in diagnosis is still needed in future research.

Advanced detection technologies and computational methods significantly increase the sensitivity and accuracy of EV‐based diagnostics.^229^ In recent years, artificial intelligence technology has also been widely applied in the diagnosis of melanoma. By using artificial intelligence strategies such as machine learning and deep learning to input Raman spectroscopy datasets into AI models, the accuracy of exosome detection and melanoma cell detection can be significantly improved.^230^ Furthermore, researchers have developed a highly sensitive detection strategy for absolute quantification of single EVs and have used this technology to detect the expression of PD‐L1 protein in melanoma‐derived single EVs. This can clearly reflect cancer progression and has the potential to become an effective tumour diagnostic biomarker.^231^

### 7.2 EVs as predictive factors

EVs hold immense promise not only as diagnostic tools but also in predicting disease progression, treatment response and patient survival^232^ (Table 4). The EMT of melanoma is one of the important reasons for the rapid progression of cancer within a year.^233^ The design of melanoma extracellular vesicles that track the EMT process can predict a patient's drug response and improve treatment outcomes. Researchers have proposed a biosensor utilizing Raman scattering and fluid dynamics, which can sensitively track EMT‐related markers of melanoma‐derived extracellular vesicles and be used for predicting targeted therapy resistance.^234^

**TABLE 4:** The clinical application of EVs in melanoma.

Clinical strategy	Target/drug	Pathway/method	Function	References
Diagnosis target	CD81 and CD63	SERS method	Provide a promising strategy for the clinical diagnosis	^223^
	PD‐L1^+^ extracellular vesicles	DEVA	Great application value in clinical diagnostic guidance	^224^
	hsa‐miR‐200c‐3p, hsa‐miR‐144‐3p and hsa‐miR‐221‐3p	‐	Serve as biomarkers for distinguishing benign melanomas from melanoma	^226^
	Y RNA	‐	A biomarker for the diagnosis of metastatic melanoma	^227^
Prognostic prediction	PD‐L1	Raman scattering and fluid dynamics	Predict targeted therapy resistance	^234^
	PD1^+^ extracellular vesicles	‐	A promising practical strategy for predicting anti‐PD1 effects	^240^
	miR‐21	Local imaging method	Predict the strength of immune therapy response	^237^
	TGF‐β	Receive immunotherapy	An important biomarker for evaluating the efficacy of melanoma immunotherapy	^109^
Therapeutic strategy	Growth hormone receptor antagonists	ABC transporter, MMP2 and N‐cadherin	Alleviate the metastasis and chemoresistance process	^253^
	MnExo@cGAMP	cGAS‐STING signal	Activate DCs and amplify CD8^+^ T cells	^255^
	Fused exosomes with Ce6 liposomes	Induce ICD	Activate DCs, enhance effector T‐cell function and increase anti‐tumour ability	^256^
	Nanovesicles containing CPT and ICG	‐	Kill tumour cells in combination with photodynamic therapy	^257^
	Fc‐modified EVs	‐	Reduce tumour burden and increase survival time	^258^
	miR‐29a‐3p	‐	Inhibit the survival and proliferation	^268^
	IL2‐ep13nsEV	MHC‐I‐related antigen presentation	Inhibit tumour migration, increase immune infiltration and enhance anti‐PD‐1 therapeutic effect	^269^, ^270^
	OVA tumour antigens	Antigen‐presenting cells	Reduce tumour recurrence and promote mouse survival	^271^
	UniSTING	IRF3/IFN‐I pathway	Change the expression of tumour‐derived exosomal miRNA	^272^
	Oncolytic extracellular vesicles	DC cells, CD4^+^ T cells and CD8^+^ T cells	Play a significant role in melanoma therapy	^273^
	Ad5/3‐D24‐ICOSL‐CD40L	NRAS mutation	Promote effector lymphocytes infiltration and exert synergistic anti‐tumour effects	^274^, ^275^
	Modified extracellular vesicle surface	TLR9 agonists	Exert anti‐tumour effects by stimulating DCs production and CD8^+^ T effector cells	^259^
	Ginger‐derived exosome	PLC and DHA	Enhance the therapeutic effect of anti‐PD‐L1	^278^
	Tumour cell membranes, nanocapsules loaded with doxorubicin	Target the tumour and lung metastasis areas	Good targeting and tumour treatment efficacy	^276^
	Exosomes loaded with hesperidin	ROS production and DNA breakage	Inhibit melanoma invasion and migration	^277^

The latest research has utilized lanthanide elements as signal amplifiers to accurately detect exosomes derived from melanoma during cancer therapy. By detecting the exosomes secreted by melanoma cells, researchers can accurately predict the immunotherapy effect of mouse melanoma models and achieve dynamic monitoring of immunotherapy in melanoma.^235^ The surface of extracellular vesicles secreted by tumour cells expresses programmed death ligand 1 (PD‐L1), and its expression can reflect the efficacy of immunotherapy.^236^ A local imaging strategy for extracellular vesicles has been developed in response to this. This strategy is capable of imaging miR‐21 in extracellular vesicles and distinguishing miR‐21‐positive regions from the overall extracellular vesicles. Subsequently, primer exchange reactions are used to amplify fluorescence signals to predict the strength of immune therapy response.^237^ For patients with metastatic melanoma, immune checkpoint inhibitor therapy is the standard treatment strategy, but biomarkers to predict the effectiveness of immune checkpoint therapy in patients still need to be identified. Researchers have detected serum exosomes from 30 melanoma patients receiving anti‐PD‐1 drug therapy, and the results showed that patients with high expression of TGF‐β in exosomes had better efficacy after receiving immunotherapy, which may become an important biomarker for evaluating the efficacy of melanoma immunotherapy.^109^ In addition, researchers have found that EVs secreted by melanoma cells can induce the transformation of peripheral blood monocytes into immune suppressive MDSCs by delivering a specific set of microRNAs (including let‐7e, miR‐99b, miR‐100, miR‐125a, miR‐125b, miR‐146a, miR‐146b and miR‐155). These miRNAs are elevated in circulating monocytes, plasma and tumour tissues of patients and their baseline levels are correlated with the clinical efficacy of anti‐CTLA‐4 or anti‐PD‐1 therapy.^238^ Although research on EV predictive biomarkers for combination therapy remains in its early stages, existing evidence from monotherapy supports their potential application in combination immunotherapy.

Serum lactate dehydrogenase (LDH) and S100B protein are the most commonly used prognostic markers, but their sensitivity and specificity are insufficient to reflect real‐time changes in the microenvironment under treatment. Information carried by EVs may hold greater predictive value.^239^ For example, a study of 110 melanoma patients revealed a notable rise in PD‐1^+^ EVs from tumour cells, alongside a significant decrease in EVs from LAG3^+^ and PD‐1^+^ T cells in those with metastatic melanoma. Monitoring the secretion of PD‐1^+^ EVs by tumours is a promising practical strategy for predicting anti‐PD1 effects, while soluble PD‐1/PD‐L1 levels do not have this predictive effect.^240^ This reveals the crucial role of EVs in dynamically monitoring immunotherapy responses. Furthermore, the levels of melanoma inhibitory protein (MIA) in exosomes not only correlate highly with serum MIA but also show a strong association between elevated exosomal MIA concentrations and shorter patient survival times.^241^ In predicting metastatic risk, serum exosomal MDA‐9 and GRP78 proteins are significantly elevated in metastatic cancer patients, potentially indicating the formation of a pre‐metastatic microenvironment earlier than conventional imaging modalities.^242^, ^243^

EV‐based liquid biopsy will be crucial in guiding future clinical decisions for melanoma, particularly in addressing resistance challenges.^244^ For patient stratification and treatment selection, EV analysis must prospectively incorporate assessment of ADC targets. Due to the important role of ADC drugs in overcoming traditional treatment resistance, it is necessary to analyze the multi‐omics profile of EVs and quantitatively analyze potential ADC targets in the future.^245^, ^246^ This will provide a basis for personalized immunotherapy, targeted therapy and the optimal combination of ADC drugs. In terms of treatment response and resistance monitoring, the resistance of EVs is influenced by downregulation of target expression, downstream signal reprogramming, drug efflux, or immune microenvironment remodelling. For example, EVs can carry non‐coding RNAs, like circular RNAs, and enhance their resistance to ADC drugs by stabilizing proteins such as HSPB1.^247^ EV status may indicate potential sensitivity of patients to specific ADC drugs that activate immune responses.^248^, ^249^ Therefore, researchers can prospectively monitor the dynamic changes in drug resistance‐associated molecular features within EVs and elucidate their regulatory mechanisms.

### 7.3 EV‐related therapeutic strategies

**FIGURE 5:**
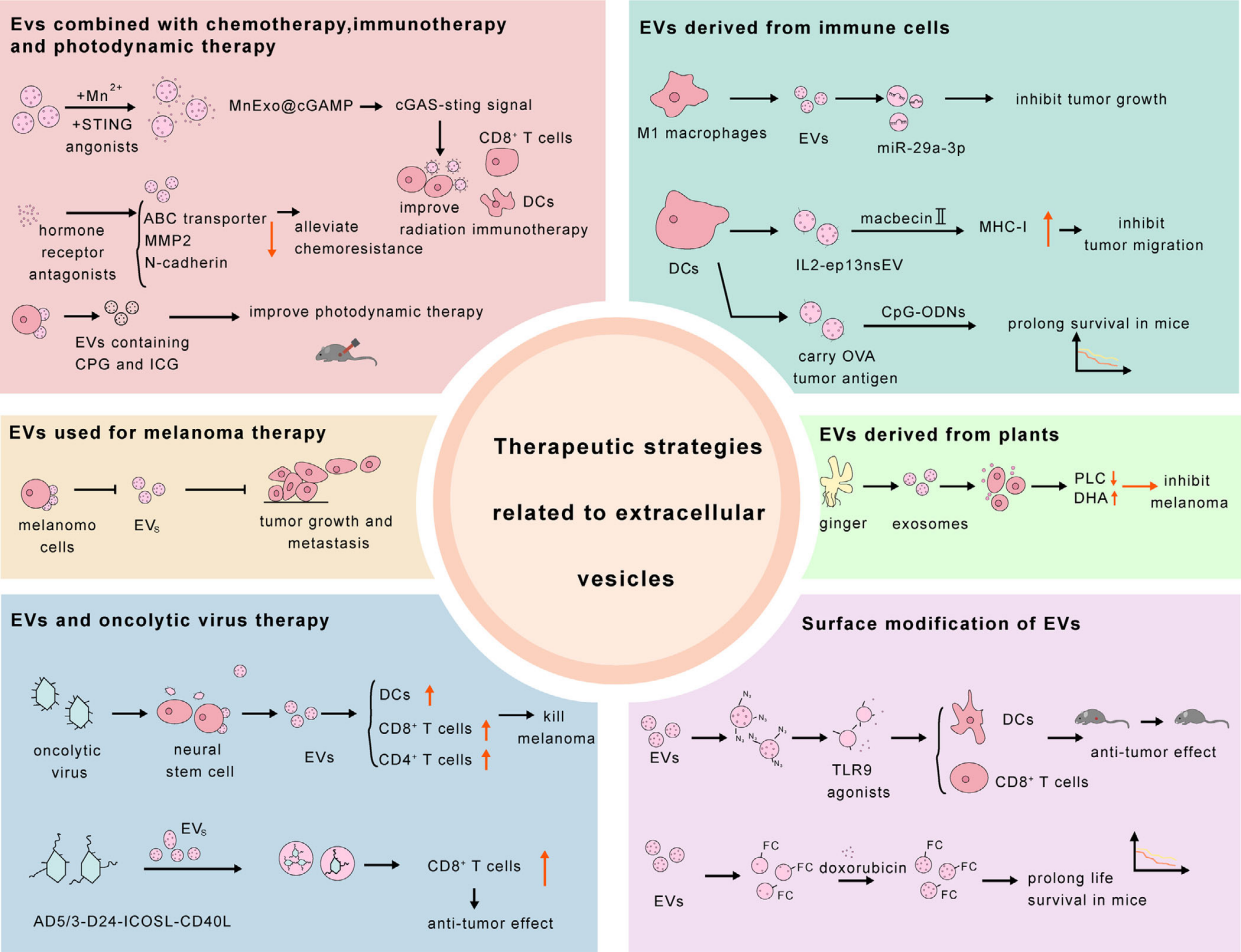
Therapeutic strategies utilizing EVs in melanoma. The melanoma treatment platform based on engineered EVs mainly includes four strategies. In immunotherapy, MnExo@cGAMP activate the cGAS‐STING pathway to enhance CD8^+^ T cell responses. M1 macrophage‐derived EVs deliver miR‐29a‐3p to inhibit melanoma progression. DCs‐derived EVs engineered with IL‐2, combined with macbecin II, improve antigen presentation. In photodynamic therapy, tumour‐derived EVs loaded with CpG oligodeoxynucleotides and ICG enhance photodynamic therapy efficacy. And photosensitive γδ‐T cell‐derived exosomes fused with Ce6 liposomes induce immunogenic cell death. In the field of targeted drug delivery, Fc domain‐modified EVs loaded with doxorubicin (DOX) target tumour cells and prolong survival. In addition, some natural vesicles can also regulate the progression of melanoma. These platforms demonstrate the multi‐functional application prospects of EVs in the treatment of melanoma.

Extracellular vesicles are considered one of the most critical cellular communication molecules and have been extensively studied to explore their functions in disease diagnosis, prediction and treatment^250^ (Figure 5). Due to the strong limitations of cancer therapy strategies, including hypoxic microenvironment, post‐treatment inflammatory response and off‐target effects, it is necessary to design new and effective treatment strategies.^251^

#### 7.3.1 Overcoming drug resistance

The growth hormone produced by the human body plays an important role in regulating melanoma exosomes and leads to the development of chemoresistance.^252^ Researchers used growth hormone to treat melanoma and found that the expression levels of ABC transporter, MMP2 and N‐cadherin were significantly upregulated in exosomes, which promoted tumour cell migration and drug resistance. The use of growth hormone receptor antagonists can significantly reduce the levels of cancer‐related proteins in exosomes and effectively alleviate the metastasis and chemoresistance process of melanoma.^253^ Malignant melanoma has strong metastatic potential and is resistant to various treatment methods. Extracellular vesicles, as an important component of intercellular communication, have crucial impacts on the mechanism of drug resistance and the malignant progression of cancer. Using the supernatant of melanoma cell culture to isolate extracellular vesicles and treat tumour cells, it was emphasized that extracellular vesicles can promote melanoma migration and slow down the therapeutic effect of BRAF inhibitors.^254^

#### 7.3.2 Combination and engineered therapies

Researchers have designed engineered cell‐derived exosomes combined with Mn^2+^ metal immunotherapy and STING agonists to improve the efficacy of radiation immunotherapy in melanoma. This nanoparticle (MnExo@cGAMP) can specifically aggregate within tumour cells and enhance the activity of the cGAS‐STING signalling pathway in conjunction with X‐rays. Subsequently, the cGAS‐STING signalling axis promotes the expression of type I interferons, activates dendritic cells and amplifies CD8^+^ T cells.^255^ To address the limitations of conventional radiotherapy and chemotherapy efficacy and mitigate side effects, photodynamic therapy (PDT) based on the release of ROS (reactive oxygen species) from photosensitizers to inhibit tumour progression has gained attention. Researchers have fused exosomes produced by γδ‐T cells with Ce6 liposomes to achieve melanoma targeting, which is capable of inducing immunogenic cell death (ICD), activating dendritic cells, enhancing effector T‐cell function and increasing anti‐tumour ability under light irradiation.^256^ Nanovesicles containing camptothecin (CPT) and indocyanine green (ICG) secreted by melanoma cells can effectively kill tumour cells in combination with photodynamic therapy. The combined treatment strategy resulted in a tumour volume 15 times smaller than the CPT treatment strategy alone.^257^ The use of antibodies with Fc‐domains to modify extracellular vesicles can enhance targeted delivery to cancer regions, while Fc‐modified EVs loaded with chemotherapy drug doxorubicin can reduce tumour burden and increase survival time of melanoma subcutaneous tumours in mice.^258^ Additionally, recent studies have utilized metabolic labelling techniques to attach azide groups to the surface of extracellular vesicles for tracking and regulating them. The modified extracellular vesicle surface can bind to TLR9 agonists and exert anti‐tumour effects by stimulating dendritic cell production and CD8^+^ T effector cells, which can achieve a survival rate of 33% in B16F10 tumour‐bearing mice and provide guidance for vaccine development.^259^

Antibody drug conjugates (ADCs) have advanced considerably in treating solid tumours like melanoma. As carriers in the TME, EVs have multiple interactions with ADC therapy, which may weaken the therapeutic effect of ADC and provide new treatment directions.^260^ EVs can affect the therapeutic effect of ADC through the antigen‐shielding effect. Research indicates that numerous ADC target antigens, including specific tumour‐associated antigens, are not only found on tumour cell membranes but are also abundantly expressed on the surfaces of EVs secreted by tumour cells.^261^, ^262^, ^263^ Within the circulatory system, antigen‐positive EVs act as molecular decoys and can competitively bind ADC antibodies with high efficiency, leading to a decrease in ADC content delivered to tumour cells and inducing drug resistance.^264^ In addition, drug sequestration also impairs the efficacy of ADC antibodies. Due to their large membrane surface area and abundant surface proteins, EVs can capture ADC antibodies in the blood through non‐specific adsorption or binding to specific receptors such as Fc receptors. This sequestration reduces drug concentration or exposure time at the tumour site, thereby diminishing therapeutic effects or altering toxicity profiles.^176^ Moreover, the ability of EVs to reshape the TME (such as promoting fibrosis and regulating vascular function) has been widely demonstrated, creating a delivery barrier and hindering the penetration of ADC antibodies within the tumour.^265^ To address the current issue of EVs diminishing the efficacy of ADC therapies, researchers have devised strategies targeting EVs in the circulatory system. They discovered that removing EVs carrying target antigens through specific filtration techniques or plasma exchange can restore sensitivity to ADC drugs in drug‐resistant tumours and eliminate the tumours.^266^ Furthermore, researchers have mitigated the inhibitory effects of ADC antibodies by developing engineered EVs. For instance, precise targeted therapy was achieved by modifying EVs with Fc‐binding domains on their surface and loading them with various therapeutic antibodies.^176^ Alternatively, synergistic therapy was realized by expressing therapeutic antibodies on the EV surface while loading them with small‐molecule inhibitors internally.^267^ These EV‐based precision delivery systems hold promise for overcoming ADC antibody inhibition, improving intra‐tumoral drug distribution and reducing off‐target toxicity.

#### 7.3.3 Immune cell‐derived EVs and oncolytic vectors

The extracellular vesicles of immune cells also have great therapeutic prospects in immune‐sensitive tumours such as melanoma. M1 macrophages usually have a pro‐inflammatory phenotype and can inhibit tumour growth. M1 macrophage‐derived extracellular vesicles were obtained using ultracentrifugation for melanoma therapy. The results showed that M1 macrophage‐derived extracellular vesicles could inhibit the survival and proliferation of melanoma cells by loading miR‐29a‐3p.^268^ Dendritic cell vesicles can also have good therapeutic effects in melanoma treatment. The combination of dendritic cell‐engineered vesicles (IL2‐ep13nsEV) and macbecin II shows that they can synergistically activate MHC‐I‐related antigen presentation, promote tumour death, inhibit tumour migration, increase immune infiltration and enhance the anti‐PD‐1 therapeutic effect in solid tumours (including melanoma and breast cancer).^269^, ^270^ Extracellular vesicles derived from dendritic cells can deliver OVA tumour antigens to similar dendritic cells, prompting antigen‐presenting cells to express the corresponding tumour antigens. And the dual effect of combining CpG‐ODNs simultaneously reduced tumour recurrence and promoted mouse survival.^271^ Furthermore, researchers have designed a STING simulator (uniSTING) to activate the IRF3/IFN‐I pathway instead of the NF‐κB signal axis, which changes the expression of tumour cell‐derived exosomal miRNAs, enhances the sensitivity of dendritic cells, promotes the production of CD8^+^ T cells and shows strong anti‐tumour effects (including breast cancer, melanoma and liver cancer).^272^ In addition, the oncolytic viruses also play important roles in the anti‐tumour treatment of melanoma. The use of capsid‐modified oncolytic viruses to infect neural stem cells can result in highly targeted and tumour‐killing oncolytic extracellular vesicles. It can promote the production of DC cells and increase the number of CD4^+^ T cells and CD8^+^ T cells, thus playing a significant role in the treatment of melanoma.^273^ In addition, an oncolytic adenovirus vaccine Ad5/3‐D24‐ICOSL‐CD40L targeting *NRAS* mutations was designed. Utilizing an extracellular vesicle delivery system to promote effector lymphocyte infiltration, it exerts synergistic anti‐tumour effects in melanoma.^274^, ^275^

#### 7.3.4 Natural vesicles

The surface of tumour cell membranes contains specific proteins and lipids, and tumour cell membrane vesicles can target specific organs and have the ability to return to the original cells. A study used melanoma B16‐BL6 cell membrane nanocapsules loaded with doxorubicin to target tumour and lung metastasis areas, and the results showed good targeting and tumour treatment efficacy.^276^ In addition, some bioactive ingredients or compounds have low water solubility and bioavailability, so delivery therapy involving exosomes loaded with insoluble compounds has attracted widespread attention. For example, hesperidin is a natural flavonoid compound, and researchers have used exosomes to encapsulate hesperidin for the treatment of melanoma.^277^ The results show that exosomes loaded with hesperidin have low cytotoxicity, while promoting ROS production, increasing DNA breakage, and inhibiting melanoma invasion and migration. Some plant‐derived exosomes, such as ginger‐derived exosome nanoparticles, can enhance the therapeutic effect of anti‐PD‐L1 by reducing PLC expression and enriching DHA, thereby improving the malignant progression of melanoma.^278^

### 7.4 Safety and clinical potential of EVs

Due to the inherent biocompatibility of EVs, engineered EVs demonstrate superior safety and low immunogenicity compared with traditional delivery vehicles in the short term. A meta‐analysis indicated a low incidence of serious adverse events (around  .7%) in EV‐based therapies, with no notable increase in risk when using allogeneic or engineered EVs.^279^ In terms of immunogenicity, engineered EVs loaded with mRNA only activate low‐level innate and adaptive immune responses, with lower intensity compared with lipid nanoparticles (LNP) or adeno‐associated viruses (AAV).^280^

EVs can synergistically enhance anti‐tumour efficacy by regulating multiple mechanisms, providing a clear theoretical basis for designing combination therapy plans for melanoma. Engineered EVs can synergistically combat cancer with traditional chemotherapy or targeted drugs.^281^ On the one hand, EVs can serve as carriers for precise targeted delivery, killing tumour cells by loading chemotherapy drugs (such as doxorubicin and gemcitabine) or targeted drugs (such as BRAF/MEK inhibitors). For example, EVs derived from mesenchymal stem cells loaded with gemcitabine exhibit stronger functions in promoting cancer cell apoptosis and killing.^282^ On the other hand, the combination of EVs and drugs can demonstrate the ability to reverse drug resistance. Engineered EVs can simultaneously deliver chemotherapy drugs and nucleic acid drugs (such as miRNA inhibitors), attack tumour cells and restore drug sensitivity.^283^ In addition, engineered EVs can also be combined with immune checkpoint inhibitors (ICI) to regulate the immune microenvironment and activate immune responses. First, EVs can directly reshape the immunosuppressive microenvironment. Engineered EVs loaded with NF‐κB inhibitors precisely regulate the communication network of immune cells, enhance anti‐inflammatory signals and enhance ICI efficacy.^284^ Second, EVs as a novel vaccine platform can synergistically expand anti‐tumour immune responses with ICI. A study shows that a fusion nanovaccine of melanoma cell membrane and bacterial EV membrane can deliver tumour antigens, thereby stimulating dendritic cell maturation and activating effector T cells.^285^ Although EVs have a synergistic effect on toxicity mechanisms and immune activation pathways with standard therapies, it is still necessary to clarify the optimal combination and timing of EVs with radiotherapy, targeted therapy and immunotherapy through preclinical and clinical trials in the future.

## 8 LIMITATIONS OF THE FIELD

Extracellular vesicles hold significant promise for melanoma diagnosis and treatment, yet their clinical application is hindered by several limitations, impeding progress in this area. EVs face technical challenges in standardization.^286^ Currently, EV separation and purification primarily rely on physical approaches, including ultracentrifugation, size‐exclusion chromatography and polymer precipitation, resulting in significant variability in recovery rates and purity. The differences in particle concentration, cargo content, and biological activity of EV products prepared in different laboratories weaken the reproducibility and reliability of clinical therapy.^287^ In addition, how to efficiently and specifically enrich EVs from tumour sources while avoiding interference from normal cellular EVs remains an unsolved challenge. Overall, it is difficult to establish clear quality control standards and potency determination methods for EV‐based therapeutic products.

Currently, long‐term risks associated with EV therapies remain challenging and uncertain. Different cell sources, culture conditions, and purification methods can lead to significant differences in EV subpopulation composition, surface protein profile and purity. The coexistence of these non‐target vesicles may trigger immunogenicity or off‐target toxicity.^288^ In addition, although engineered EVs improve targeting and transfer efficiency, they may alter the natural membrane characteristics or stability of EVs, which may affect their recognition, clearance and long‐term fate in vivo.^289^ Furthermore, existing clinical trials generally have a short duration and lack long‐term safety evaluations. Will prolonged or repeated administration induce immune tolerance or generate neutralizing antibodies? Could engineered EVs and their cargo accumulate in specific organs, leading to delayed toxicity? These issues still exist in long‐term clinical safety monitoring and require further exploration through relevant research.

Additionally, the lack of basic biological understanding and clinical evidence of extracellular vesicles limits their precise application. We still lack insight into the molecular mechanisms by which tumour cells dynamically regulate EV cargo sorting in response to microenvironmental signals, as well as how specific receptor cells selectively internalize different EV subtypes (such as exosomes, micro‐vesicles and melanosomes) in vivo. This uncertainty in mechanisms creates a bottleneck for the theoretical design of engineered EVs. In terms of preclinical models, most existing studies rely on immunodeficient mouse models, which cannot simulate the complex interactions between the human immune system and tumour EVs.^290^ In terms of clinical research, although some early clinical trials have demonstrated the therapeutic effect of EVs, most studies have small sample sizes, short follow‐up times or lack rigorous control group designs. Currently, there is still a serious lack of large‐scale, multi‐centre and randomized controlled phase III clinical trials related to EVs (Table 5). Furthermore, there is a lack of relevant clinical research data on how to optimize the sequence combination of EV therapy with existing radiotherapy, targeted therapy and immunotherapy.

**TABLE 5:** The clinical experiments related to EVs.

Tumour type	NCT Number	Status	Enrolment	Trial phase
Melanoma	NCT05744076	Completed	150	‐
Melanoma	NCT02310451	Completed	15	Not applicable
Lung cancer	NCT04529915	Completed	470	‐
Lung cancer	NCT03830619	Completed	1000	‐
Non–small cell lung cancer	NCT02890849	Completed	60	Not applicable
Non–small cell lung cancer	NCT01159288	Completed	41	Phase 2
Prostate cancer	NCT02702856	Completed	2000	‐
Colorectal cancer	NCT04394572	Completed	80	‐
Cholangiocarcinoma	NCT06381648	Completed	190	‐
Oral cancer	NCT06469892	Completed	225	‐
Head and neck cancer	NCT01668849	Completed	60	Phase 1
Osteosarcoma	NCT05101655	Completed	60	‐
Gastric cancer	NCT06342427	Completed	809	‐
Thyroid cancer	NCT03488134	Completed	74	‐
Breast cancer	NCT05955521	Ongoing	200	Not applicable

*Note*: The data was obtained from www.clinicaltrials.gov.

## 9 PROSPECTS

**FIGURE 6:**
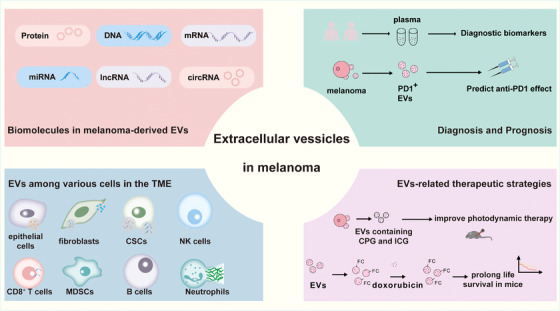
Summarize the multifaceted roles of EVs in melanoma. Melanoma‐derived EVs carry diverse biomolecules (such as proteins, DNA and mRNAs) that mediate intercellular communication within the TME. EVs reshape the TME by modulating immune cells (including T cells, macrophages, MDSCs and NK cells), stromal cells and endothelial cells, thereby promoting immune escape and metastasis. These molecular and cellular mechanisms provide the basis for clinical applications of EVs as diagnostic biomarkers, prognostic predictors and therapeutic platforms.

EVs play a crucial and complex role as key mediators of intercellular communication in the occurrence, development and treatment resistance of highly heterogeneous skin melanoma.^291^ This review systematically summarises the multiple aspects of EVs, including biological molecule carrying, immune microenvironment regulation and clinical translational applications, revealing their significant importance in the field of melanoma research. EVs can promote tumour progression and aid in immune escape. Due to their unique biological characteristics, they have become potential disease monitoring biomarkers and precise strategies for innovative treatments (Figure 6).

EVs derived from melanoma form a dynamic molecular information database through their abundant cargo, including proteins, DNA, mRNA and various non‐coding RNAs (such as miRNA, lncRNA and circRNA), which reflect the pathological status of parental cells and the regulatory mechanisms of tumour malignant progression. For example, the low expression of Cx43 protein in EVs is associated with poor prognosis in patients, while the high expression of PD‐L1 directly mediates T cell dysfunction. At the nucleic acid level, the mutated DNA carried by EVs (such as *BRAF* V600E) provides a convenient source for liquid biopsy. In addition, the lncRNA Gm33149 carried by EVs can activate the Wnt pathway and promote tumour metastasis by competitively adsorbing miR‐5623‐3p, while circRPS5 exhibits the potential to inhibit tumours. These findings establish the dual identity of EVs as molecular targets and messengers of information in melanoma, and the changes in their contents depict the evolutionary trajectory of melanoma.

EVs are core molecules that reshape the TME and drive immune suppression. Melanoma cells communicate bidirectionally with almost all components of the TME by releasing EVs, and shape an ecological niche that facilitates tumour growth and metastasis. At the immune level, EVs can deliver PD‐L1 molecules to directly induce T cell ageing, and can also affect astrocytes and promote the release of inflammatory factors by loading molecules such as miR‐146a‐5p. In addition, EVs can promote macrophage polarization towards the M2 phenotype, expand myeloid‐derived suppressor cells (MDSCs) and enhance the function of regulatory T cells (Tregs), thereby synergistically establishing a robust immunosuppressive barrier. At the stromal level, EVs can transform normal fibroblasts into cancer‐associated fibroblasts (CAFs), stimulate angiogenesis and alter the function and permeability of endothelial cells, providing a pre‐metastatic microenvironment for distant tumour metastasis. Intervening in the generation, secretion, or function of EVs can affect the dynamic remodelling of TME, making it an attractive strategy for reversing immune suppression and overcoming drug resistance.

The detection of EVs based on liquid biopsy has shown significant advantages in diagnosis and prognosis prediction. Non‐invasive and dynamic monitoring of melanoma can be achieved by detecting specific proteins (such as PD‐L1), nucleic acid mutations, or non‐coding RNA profiles of EVs in body fluids such as plasma. For example, high expression of TGF‐β mRNA in extracellular vesicles is associated with a good response to anti‐PD‐1 immunotherapy, while the level of PD‐L1^+^ EVs from tumour sources may become a biomarker for predicting the efficacy of immunotherapy. However, the accuracy of EV‐based detection also faces some issues. Multiple types of cells (including tumour cells, immune cells, stromal cells, etc.) in the TME secrete large amounts of EVs, resulting in high heterogeneity of EVs in the circulatory system. Only detecting markers of total EVs in the circulatory system may be interfered with by a large number of normal tissue‐derived EVs, thereby reducing the sensitivity of tumour‐specific markers (such as mutant DNA and cancer‐associated proteins) detection. Moreover, the physical characteristics of TME, such as vascular density and permeability, can affect the release efficiency of EVs from distinct sources and lead to differences in the composition of EVs between blood and tumours. Meanwhile, immunotherapy or spontaneous tumour necrosis can significantly alter the state of TME and alter the secretion and composition of EVs in the short term, thereby causing dynamic interference to detection at different time points. It is necessary to incorporate TME features into the development of EV biomarkers to promote the clinical application of EV liquid biopsy. In the field of therapy, the natural biocompatibility, low immunogenicity and targeting ability of EVs make them a good drug delivery nanoplatform. Researchers have successfully loaded chemotherapy drugs, small interfering RNA (siRNA), mRNA vaccines, or immune agonists (such as STING agonists) into EVs through engineering modifications, achieving precise targeting of tumours. For example, dendritic cell‐derived exosomes loaded with BRAF siRNA, tumour cell membrane vesicles displaying specific antigens and EVs modified with chimeric antigen receptors have all shown significant anti‐tumour effects in preclinical models. In addition, EVs derived from natural killer (NK) cells, M1 macrophages, or dendritic cells have strong immune activation or direct tumour killing abilities, providing new methods for cell therapy.

Currently, there are still many challenges in the application of EVs. The intrinsic molecular mechanisms underlying the biogenesis, secretion and uptake of EVs by target cells require further exploration to provide new targets for the development of intervention strategies. EVs have high heterogeneity, and their separation, purification and standardized analysis face severe technical challenges. Obtaining high‐purity and specific subgroups of EVs is an important prerequisite for ensuring their application. In addition, the drug loading efficiency, in vivo targeting, long‐term safety and potential immunogenicity of engineered EVs still need to be evaluated. Moreover, combining EVs with existing standard therapies such as immune checkpoint inhibitors, targeted drugs and radiotherapy to develop personalized treatment plans is a direction that needs to be further explored in the future.

## AUTHOR CONTRIBUTIONS

Junshu Li and Wencheng Zhou jointly drafted the manuscript. Ying Cen and Yong Qing designed and revised the manuscript. All authors have reviewed and approved the final manuscript.

## FUNDING

This work was supported by the Sichuan Science and Technology Program, China Program (grant number: 2023YFS0064), the Postdoctor Research Fund of West China Hospital, Sichuan University (grant number: 2025HXBH094) and the Sichuan University Interdisciplinary Innovation Fund.

## ETHICS STATEMENT

Not applicable.

## CONFLICT OF INTEREST STATEMENT

The authors declare no conflict of interest.

## Data Availability

No data were used for the research described in the article.
